# Preference for Fractal-Scaling Properties Across Synthetic Noise Images and Artworks

**DOI:** 10.3389/fpsyg.2018.01439

**Published:** 2018-08-29

**Authors:** Catherine Viengkham, Branka Spehar

**Affiliations:** Department of Psychology, University of New South Wales, Sydney, NSW, Australia

**Keywords:** esthetics, fractal dimension, art, preference, perception, fractal-scaling, complexity

## Abstract

A large number of studies support the notion that synthetic images within a certain intermediate fractal-scaling range possess an intrinsic esthetic value. Interestingly, the fractal-scaling properties that define this intermediate range have also been found to characterize a vast collection of representational, abstract, and graphic art. While some have argued that these statistic properties only serve to maximize the visibility of the artworks’ spatial structure, others argue that they are intrinsically tied to the artworks’ esthetic appeal. In this study, we bring together these two threads of research and make a direct comparison between visual preference for varying fractal-scaling characteristics in both synthetic images and artworks. Across two studies, viewers ranked and rated sets of synthetic noise images and artworks that systematically varied in fractal dimension for liking, pleasantness, complexity, and interestingness. We analyzed both average and individual patterns of preference between the two image classes. Average preference peaked for intermediate fractal dimension values for both categories, but individual patterns of preferences for both high and low values also emerged. Correlational analyses indicated that individual preferences between the two image classes remained moderately consistent and were improved when the fractal dimensions between synthetic images and artworks were more closely matched. Overall, these findings further support the role of fractal-scaling statistics both as a key determinant of an object’s esthetic value and as a valuable predictor of individual differences in esthetic preference.

## Introduction

Since the introduction of fractal geometry by Mandelbrot (1977), interest in the universality and esthetic appeal of fractal-like statistics has taken many forms. At their core, fractals are patterns characterized by repeating spatial characteristics at increasingly fine scales (Mandelbrot, 1977; Forsythe et al., 2011). The most visually recognizable examples are exact mathematical fractals, such as Koch’s snowflake or the Sierpinski triangle. The key feature is that any portion of these fractal patterns, when magnified in scale, will appear identical to the whole pattern. However, many objects in the natural world are also characterized by a fractal geometry, for example, the outline of a cloud or the shape of a coastline. In contrast to exact fractal patterns, the spatial structures of natural objects, textures, and scenes are statistical fractals and contain a degree of randomness. Statistical fractals are typically similar at different levels of magnification, but not necessarily identical. Consequently, statistical fractals look alike across different spatial scales in regard to their spatial qualities, such as roughness, density, or complexity.

The structure of a fractal pattern can be quantified using a measure called fractal dimension (D). Fractal dimension relates the amount of spatial structure occurring at different levels of magnification to the overall structure. More specifically, D increases as the overall structure increases in fine spatial detail (Mandelbrot, 1982). As such, fractal dimension is related to other scaling techniques that characterize the ratio of coarse to fine spatial structure in images, such as the 1/f^α^ amplitude spectrum. Whereas the slope α of the 1/f^α^ amplitude spectrum quantifies the relationship between the amplitude of luminance variations across low to high spatial frequency variations, fractal dimension does this with structural density and the two are inversely related. Importantly, both these measures of fractal-scaling statistics have been adopted as a means for objectively quantifying one of the central elements in empirical esthetics – visual complexity (Pentland, 1984; Cutting and Garvin, 1987).

Since the past century, complexity has been a central pillar in our understanding of beauty and esthetic appreciation. In his seminal study, Berlyne (1971) manipulated the complexity of different visual patterns and shapes by changing parameters related to their order, heterogeneity, regularity, and numerosity. On the basis of his findings, he suggested that the relationship between preference and complexity followed an inverted U-curve, in which preference peaked at the point where optimal arousal is achieved. Since this proposal, the point of optimal complexity (in which an object is regarded as neither too simple nor too overstimulating) has been the subject of extensive investigation and empirical scrutiny.

Subsequent studies have tackled the difficult task of quantifying and manipulating complexity in many unique ways. Generally speaking, methods of selecting specific parameters (e.g., number of elements in a pattern or number of sides in a polygon) and changing them as a direct manipulation of complexity have been the most typical (Forsythe et al., 2011). For example, Güçlütürk et al. (2016) generated geometric patterns using a computer algorithm that allowed them to precisely predefine the stimuli’s complexity as a function of decreasing shape size and rate at which the shapes filled a space. Such flexibility has allowed complexity to be variously defined across an equally vast array of visual objects, such as line drawings (Vitz, 1966), random polygons (Aitken, 1974; Martindale et al., 1990), grid textures (Ichikawa, 1985; Jacobsen, 2004; Friedenberg and Liby, 2016), abstract patterns (Gartus and Leder, 2017), and so forth. However, the lack of a consistent quantification has prevented researchers from reaching a definitive consensus regarding the place of complexity in esthetic appreciation (Nadal et al., 2010; Forsythe et al., 2011).

Measures of fractal-scaling characteristics, such as fractal dimension and amplitude spectrum slope (α), afforded researchers additional flexibility and objectivity in the types of stimuli through which they could explore esthetic complexity. Cutting and Garvin (1987) were one of the first to demonstrate how perceived complexity increased with increasing amounts of fine structure in exact, mathematical fractal patterns. Since then, fractal dimension has been used as a measure of complexity across a vast range of visual stimuli including photographs (Spehar et al., 2003), paintings (Taylor et al., 1999; Redies et al., 2007a; Graham and Redies, 2010; Hayn-Leichsenring et al., 2017; Redies and Brachmann, 2017), landscape silhouettes (Hagerhall et al., 2004), and a wide variety of computer-generated images (Aks and Sprott, 1996; Spehar et al., 2016).

Overall, these studies have revealed remarkably robust relationships between fractal-scaling statistics, complexity, and esthetic preferences. For example, we previously found that average esthetic preferences consistently peaked at the intermediate fractal dimension values across a wide array of image types ranging from grayscale, two-tone, edge, and terrain variations (Spehar et al., 2016). Even though these images were visually distinct in appearance, the study revealed a remarkable degree of within-subject consistency in preferences for specific fractal dimensions across stimuli. This suggests that low-level sensitivities to fractal-like statistics may have a predictable and contextually consistent impact on our esthetic choices. Through this, fractal dimension has also allowed for a greater understanding of the nature and course of individual variability in esthetic preference for complexity (Güçlütürk et al., 2016; Spehar et al., 2016; Street et al., 2016).

It is likely that this preference intimately links to the pervasive presence of fractal-scaling statistics in both natural scenes and art. That is, intermediate values of fractal dimension also broadly define the range occupied by most natural scenes (Tolhurst et al., 1992; Taylor and Spehar, 2016). Natural scenes are a constant part of our daily landscape and possess the same spatial characteristics to which our visual systems are the most sensitive (Knill et al., 1990; Spehar et al., 2015), coinciding with the increased discrimination ability in this range (Malo et al., 1997; Spehar et al., 2016). As a result, extra amount of bits is required in this range to obtain pleasing images (Malo et al., 2000), which is consistent with the connection between natural image statistics and perceived image quality (Moorthy and Bovik, 2011; Saad et al., 2012). Interestingly, these spatial characteristics appear to be the most esthetically appealing (Spehar et al., 2015), and this same intermediate range characterizes a vast array of artworks (Taylor et al., 1999; Redies et al., 2007b; Graham and Field, 2008). Comprehensive analyses of the statistical properties of different genres of art reveal that artists consistently gravitate toward the same intermediate fractal-scaling range occupied by natural scenes (Redies et al., 2007a,b; Mather, 2014). While it may be argued that artists are purposefully attempting to replicate the statistical properties of natural scenes to maximize realism, a large number of abstract and non-representative works also fall within this intermediate D range (Redies and Brachmann, 2017). As a result, researchers argue that artists’ esthetic choices for producing intermediately complex art is a consequence of our visual systems’ intrinsic sensitivity and attraction to fractal statistics in the natural scenes range (Graham and Field, 2007; Redies, 2007; Spehar et al., 2015).

While these findings implicate a close relationship between fractal dimension and esthetic preference in art, investigations so far have predominantly been descriptive. In other words, they have been limited to measuring the statistical properties of art itself rather than the subjective responses of individual observers. Paintings falling in the intermediate D range may not necessarily always be the ones observers find the most attractive. Furthermore, while many artists choose to reflect intermediate fractal-scaling properties in their art, individual differences in artistic choices have also emerged. For example, Jackson Pollock’s work is uniquely characterized by its extreme detail and complexity, subsequently having measured D values far greater than the average abstract artwork (Taylor et al., 1999, 2011). So far, individual differences in the esthetic preference for fractal-scaling properties have mainly been investigated in synthetic fractal images. We know that in the context of synthetic images, subjects typically prefer fractal dimension values in the intermediate range, but persistent preferences for both higher and lower degrees of complexity have always arisen (Redies et al., 2007a,b; Spehar et al., 2016; Redies and Brachmann, 2017). These preferences appear to remain relatively consistent between and within individuals even across the vastly different variations of synthetic fractal stimuli being tested. However, we have yet to determine whether individual preferences in fractal-scaling statistics extend from synthetic images to real-world art, which also have natural variations in fractal-scaling characteristics.

This study investigates whether preference patterns for specific fractal-scaling characteristics remain consistent both inter- and intraindividually across both synthetic fractal images and real-world art. Our interest in the stability of individual differences in preference across different types of images was one of the key factors motivating this research. For this reason, we chose to run this study online in order to be able to get access to a more diverse pool of subjects from relatively different backgrounds and demographics compared to the standard pool of university students.

Both advantages and reservations about using an online recruitment platform in perception research have been extensively discussed (Woods et al., 2015). However, we saw this approach as an opportunity to assess whether the same relationship between fractal-scaling and visual preference obtained under controlled conditions in the laboratory can be reproduced online despite the absence of display calibration and color rendering. As mentioned, the general findings from our laboratory studies, conducted in settings where we could ensure that viewing conditions were consistent during experiments and across participants, were highly robust. Therefore, the current study adopted the same synthetic fractal images and fractal-scaling variations as those in our previous studies to attempt to replicate the effect (Spehar et al., 2003, 2015, 2016). In addition, to extend our previous findings with the synthetic images to real-world art, we also use a subset of paintings that are matched in their fractal-scaling characteristics to the synthetic images.

We predict that, on average, most individuals will prefer synthetic fractal images and paintings with fractal dimensions in the intermediate range. However, congruent with previous studies, we also predict that clusters of individuals with preferences for lower and higher fractal dimensions will also emerge (Spehar et al., 2016). We predict that these preferences will be consistent across different types of synthetic fractal images and across both representational and non-representational artistic stimuli.

## Experiment 1: Materials and Methods

### Design

The study adopted a 10 × 3 within-subjects design. The first variable, image type, consisted of 10 categories: three types of synthetic fractal images (grayscale, edges, and thresholded) and seven types of paintings (abstract, buildings, flowers, forests, mountains, rivers, and seas). Secondly, all image categories varied on three levels of fractal dimension: low, intermediate, and high. Four outcomes were measured: preference, pleasantness, complexity, and interest.

### Participants

A total of 184 participants were recruited via the online recruitment platform Amazon Mechanical Turk^1^. Due to the online nature of the experiment, all participants were asked to read an online information statement prior to the study and indicate their consent. They were reimbursed with US$4.50 for taking part in the study. All experimental procedures were approved by the UNSW Human Research Ethics Advisory Panel (UNSW HREAP-C).

Prior to analysis, data attained from the online experiment were manually filtered for quality and proportion of completion. Specifically, data from subjects who clearly failed to complete the task, neglected instructions, or showed an explicit indifference to task requirements were excluded from the final analysis. As a result, 13 subjects were excluded and of the remaining 171 subjects: 29.2% were female and the mean age was 33.15 years. A total of 65% indicated their country of residence as the United States, 24% as India, and 7% as the United Kingdom.

### Materials and Apparatus

#### Synthetic Fractal Images

**FIGURE 1:**
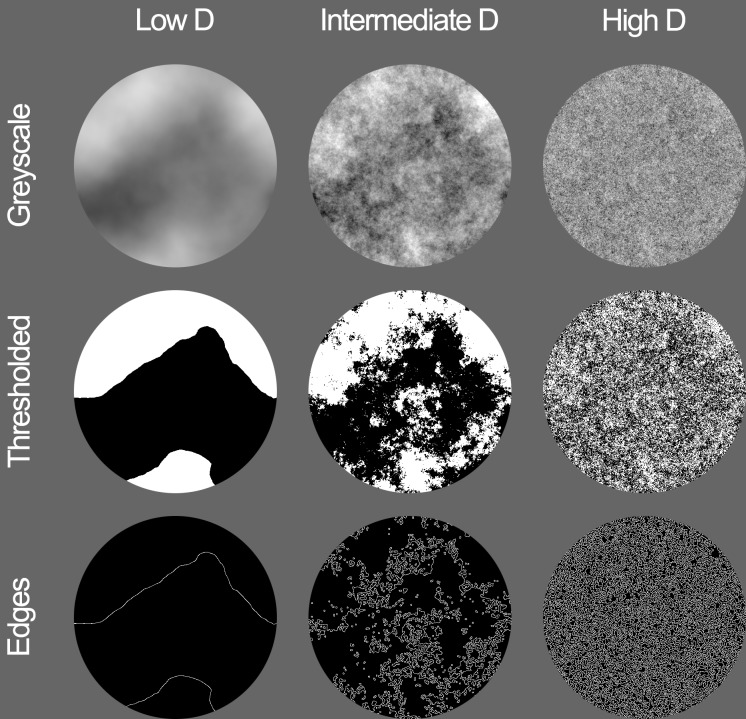
Synthetic fractal stimuli used for Experiment 1. Image types shown from top to bottom are grayscale, thresholded, and edges with the average fractal dimension D of 0.978 (left), 1.710 (middle), and 2.030 (right), respectively.

The examples of three different types of synthetic fractal images used in this study are depicted in **Figure 1**. The top to bottom rows depict grayscale, thresholded, and edges image types, respectively. The columns depict low (left), intermediate (middle), and high (right) fractal dimension variants of the three different image types.

To produce the grayscale synthetic fractal images, we used MATLAB to generate a grayscale pattern of 512 × 512 pixels with each pixel value (0–255) selected from a Gaussian distribution. A Fourier transform was then performed to create amplitude frequency spectra with three different levels of 1/f^α^ falloff, given by amplitude spectrum slopes (α) of 0.5, 1.2, and 2.3. The mean brightness and the RMS contrast of grayscale images were controlled at 126 and 0.30, respectively. Following these specifications, four different seeds of grayscale images were created, resulting in a total of 12 grayscale fractal images (4 seeds × 3 amplitude spectrum slope values). These images were then converted into their thresholded (black and white) counterparts by binarizing the grayscale image at the mean luminance level, such that pixels below mean luminance were assigned as black and those above as white. Edge-only images were created by extracting the edges from the thresholded black and white images (see Spehar et al., 2016). Altogether, these variations resulted in 36 different images in total.

For the purposes of this study, the amplitude spectrum manipulations allowed us to generate synthetic fractal images with low, intermediate, and high values of D. We used a box-counting technique to calculate the corresponding fractal dimension of the images with different amplitude spectra. The box-counting method divides the image into a grid of equally sized squares or “boxes” and counts how many are occupied by the pattern in the image. This process is repeated for smaller and smaller box sizes, which functions to capture more of fine structure of the patterns. For paintings and natural scenes, this relationship between box size and occupation follows a power law. The slope of the log–log plot of this relationship gives the fractal dimension, and the average measured D values for the synthetic images of each slope were 2.02, 1.63, and 0.98, respectively (see **Table 1**). These values are in good agreement with the empirically derived conversion values reported previously (Spehar and Taylor, 2013; Bies et al., 2016).

**Table 1:** Average fractal dimension and luminance values for each category of paintings and each type of synthetic fractal image in Experiment 1.

	Average fractal dimension			Average luminance		
	Low	Intermediate	High	Low	Intermediate	High
Abstract	1.06	1.42	1.85	147.1	115.3	119.0
Buildings	1.18	1.45	1.67	128.5	118.2	126.1
Flowers	1.29	1.42	1.72	76.80	68.86	80.15
Forest	1.13	1.40	1.73	127.6	114.72	102.0
Mountain	1.08	1.37	1.65	141.3	111.8	121.5
River	1.14	1.44	1.69	124.5	89.17	83.73
Sea	1.18	1.40	1.70	128.2	147.1	148.4
Mean	1.154	1.418	1.711	124.8	109.3	111.6
Grayscale	0.97	1.62	2.05	127.5	127.5	127.5
Threshold	0.97	1.62	2.05	115.3	123.7	127.6
Edge	0.99	1.66	1.97	1.530	27.27	74.51
Mean	0.98	1.63	2.02	81.46	92.81	109.9

#### Paintings

Paintings were sourced from multiple collections such as the Google Art Project and the JenAesthetics database (Amirshahi et al., 2015). In total, we collected 1076 paintings (138 abstract, 162 buildings, 186 flowers, 174 forests, 134 mountains, 170 rivers, and 112 seascapes). Western-style oil paintings formed the majority of the database. Luminance, fractal dimension, and amplitude spectrum slope were measured for every painting. All three measures were taken based on the grayscale versions of the images. The mean value of the grayscale image was taken as a luminance.

Separately for each category, paintings were ordered from high to low in both D and α. We partitioned paintings with D > 1.65 into the “High D” group, paintings with 1.3 < D < 1.50 into the “Intermediate D” group, and paintings with D < 1.20 into the “Low D” group. In total, 123 paintings were used for the study (12 abstract, 18 buildings, 18 flowers, 18 forests, 21 mountains, 21 river, and 15 seas), resulting in 45 images per level of D (to view all paintings, see **Supplementary Images**). Average fractal dimension and luminance values for each of the painting categories are shown in **Table 1**. An analysis of variance (ANOVA) was performed to ensure that the average D values of each fractal dimension group differed significantly [*F*_(6,102)_ = 664.851, *p* < 0.01].

Average luminance was found to vary significantly across painting category [*F*_(6,102)_ = 9.451, *p* < 0.01] and marginally across fractal dimension [*F*_(2,102)_ = 3.218, *p* < 0.044]. However, there were no significant interactions between the two [*F*_(12,102)_ = 0.951, *p* = 0.50]. This meant that painting categories with markedly lower or higher than average luminance (e.g., flowers) shared these characteristics across all levels of fractal dimension. While the differences in average luminance may not be optimal, we aimed to retain as much of paintings’ original appearance as possible. Because luminance did not significantly differ across fractal dimension within individual painting categories, we regard this as an acceptable compromise to maintain the original values of the artworks.

**FIGURE 2:**
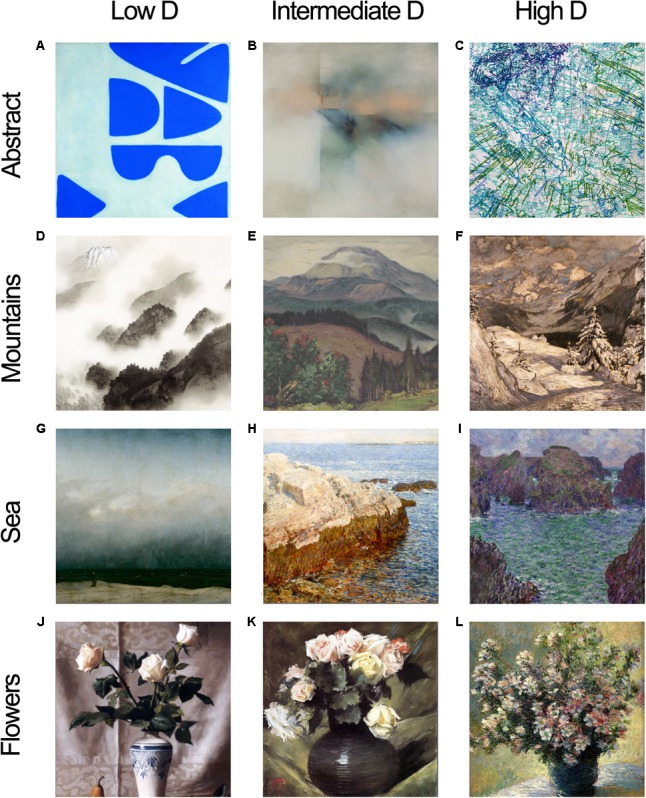
Examples of matched triads from four painting categories. Abstract paintings: **(A)** William Scott, Berlin Blues 6, 1966, **(B)** Fernando Zóbel de Ayala y Montojo, Flight in Pink, 1966, **(C)** Ingrid Calame, Untitled (Traces 1, 2, and 3), 2006. Mountain paintings: **(D)** Yokoyama Taikan, Mountain after a Shower, 1940, **(E)** Martin Benka, Choè Mountain, **(F)** Filippo Carcano, In Midwinter, 1909. Sea paintings: **(G)** Caspar David Friedrich, The Monk by the Sea, 1809, **(H)** Childe Hassam, Cliff Rock – Appledore, 1903, **(I)** Claude Monet, Port Goulphar, 1887. Flower paintings: **(J)** Maureen Hyde, Still Life with White Roses, **(K)** William Merritt Chase, Flowers (aka Roses), 1888, **(L)** Claude Monet, Vase of Flowers, 1882. Images increase in fractal dimension from left to right. Samples shown are cropped to squares, but original aspect ratios of paintings were used in the study.

Furthermore, to ensure that preference judgments were made primarily considering the fractal dimension, we created triads of artwork images that were matched in the overall color tone, content, and luminance within each painting category. For example, the 12 abstract paintings were divided into four triads such that the three paintings within a single triad all possessed similar colors and content and differed only in the fractal dimension (**Figure 2**).

### Procedure

The experiment was coded using the Inquisit 5 Web experimental software and accessible via the Inquisit 5 Web Player, which subjects downloaded onto their devices before beginning the study. On launch, the Inquisit Web Player covered the entire screen of the subject’s device. A maximum display area of 1280 × 800 pixels was preset to ensure consistent image positioning across screens and such that the width of any image would only cover their respective areas and not overlap with adjacent images. This also ensured that images were not susceptible to vast changes in size regardless of the resolution of the monitor on which they were being viewed. The experiment could not be performed on a tablet or mobile devices as it required mouse clicks to record responses. Of the 171 subjects, 92.4% (*n* = 158) completed the experiment on a Windows OS and 7.6% (*n* = 13) completed it on a Mac OS.

The study consisted of two parts. Part 1 investigated preferences for paintings and synthetic images using an adapted 3-alternative forced-choice (3AFC) preference task. Part 2 further examined subjective evaluations of the same images using bipolar rating scales. After reading and consenting to the information statement and answering basic demographic questions, Part 1 of the study began.

#### 3AFC Preference Task

On each trial, images from one triad (described in *Materials and Apparatus*) were displayed in a row across the screen. Participants were prompted to click with a cursor on their favorite image out of the three choices. The chosen image was then removed, and participants were prompted to click on their next preferred choice out of the remaining two. This method provided an efficient and seamless way of ranking images in order of preference and was repeated for all the image triads in the same manner. These triads were randomly presented within blocks of the same image category. The three blocks of synthetic fractal images were completed first, followed by the seven blocks of paintings. The position of images from each fractal dimension category was randomized for each trial.

In total, the participants evaluated 12 trials with synthetic noise images (4 grayscale, 4 thresholded, and 4 edges) and 41 trials with paintings (4 abstract, 6 buildings, 6 flowers, 6 forests, 7 mountains, 7 rivers, and 5 seas). The differences between the number of trials per painting category were due to the number of matched triads that were possible to create from our database of low, intermediate, and high D paintings for each category. Some categories simply had fewer viable matches compared to others.

#### Ratings Task

Part 2 immediately followed the completion of Part 1. Participants were asked to rate individual paintings on three measures: pleasantness, complexity, and interestingness. Measures were collected on 7-point semantic differentiation scales with bipolar adjectives on opposite sides: pleasant–unpleasant, simple–complex, and boring–interesting. Points on the sliders were centered by default and participants moved each slider in response to the single image presented on a given trial. A total of six images (two low, two intermediate, and two high D) were randomly selected from each category of paintings and synthetic fractal images. In total, participants rated 42 paintings and 18 synthetic images on each of the three measures.

After completing the study, subjects were debriefed and redirected from the Inquisit Web Player to their web browser where they received their completion code. In total, the study took approximately 30 min, and subjects were allowed up to an hour to complete it and submit their completion code in MTurk.

## Experiment 1: Results

### 3AFC Preference Task

Preference data were collated such that, for each image, the count of the number of times they were chosen as the first preference was multiplied by two and then summed with the count of times they were selected as the second preference. This resulted in a matrix of aggregate preference scores for the low-, intermediate-, and high-fractal-dimension stimuli across each of the image groups (grayscale, edge, and threshold synthetic noise images and all of the paintings). Raw counts were recalculated as “proportion of times chosen” by taking the count of times images in a particular fractal dimension category were selected and dividing this by the total number of selections made for that image category. Higher proportions indicated greater preference.

#### Population Preferences for Images With Low, Intermediate, and High Fractal Dimension

**FIGURE 3:**
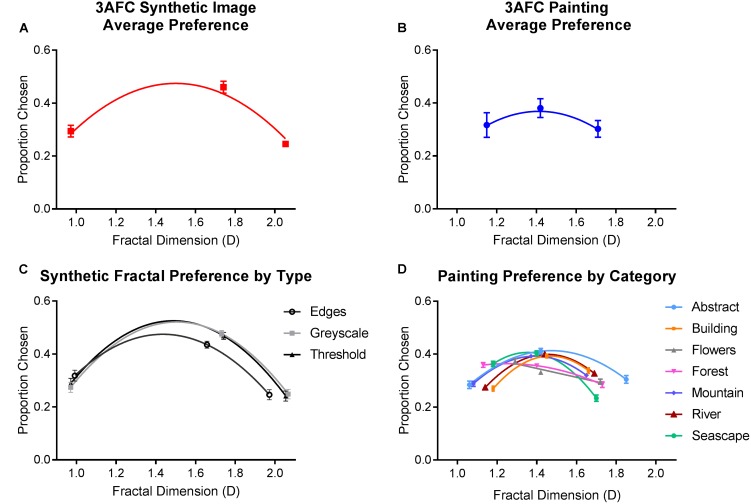
Average preferences from Experiment 1, where a higher proportion chosen indicates a greater preference. Top row depicts the average (and SE) proportion of times **(A)** synthetic and **(B)** painting from each level of fractal dimension were selected. Bottom row shows the average (and SE) proportion of times each category within the **(C)** synthetic and **(D)** painting image types was chosen, plotted as a function of their measured fractal dimension.

Analyses of population preferences were conducted separately for synthetic and painting stimuli. Average preferences are plotted in **Figure 3**. Repeated-measures ANOVAs were employed in both cases, with fractal dimension (low, intermediate, and high) and the image category (abstract, building, flowers, forests, mountains, rivers, and sea for paintings; grayscale, threshold, and edge for synthetic) as the independent variables. Sphericity assumptions were violated in both analyses; therefore, our statistical outcomes are reported with the Greenhouse-Geisser correction applied.

For synthetic images, we found a significantly greater average preference for images with intermediate D (46% of choices) compared to low (29.4%) and high (24.6%) D [*F*_(1.554,264.239)_ = 40.755, *p* < 0.01]. This preference was consistent within each image category (grayscale, threshold, and edge) and did not differ significantly between them [*F*_(1.983,337.178)_ = 0.90, *p* = 0.912]. However, a marginally significant interaction [*F*_(2.902,493.418)_ = 2.408, *p* = 0.048] indicated that the peak in preference for intermediate D was slightly lower, and preference for low D higher, in synthetic edge images compared to the other two variations.

For paintings, intermediate D paintings accounted for 37.8% of choices and this was significantly higher compared to low (30.5%) and high (31.7%) D images [*F*_(1.475,250.823)_ = 42.848, *p* < 0.01]. Analysis also revealed a significant interaction between fractal dimension and painting category [*F*_(12, 1512.194)_ = 17.536, *p* < 0.01]. Intermediate D images were preferred in abstract, building, mountain, river, and seascape paintings but not in flower and forest paintings, in which low D images were more highly preferred.

#### Internal Consistency in Preference for Different Fractal Dimensions Across Image Types

To determine whether individual participants made consistent preferences for either low-, intermediate-, or high-fractal-dimension images across different synthetic and art images, we calculated the pairwise correlations between preferences for different image types for each subject. This was done for every combination of either synthetic or art image types, as well as for overall synthetic preferences (averaged across grayscale, thresholded, and edge images) and painting preferences (averaged across seven different painting types). These combinations totalled 56 unique pairwise correlations per subject, which were then each averaged across the 171 subjects and are shown in **Supplementary Figure 1**.

On average, preference between different image categories for individual subjects ranged from being moderate to highly correlated. In particular, preferences between different types of synthetic fractal images were highly correlated ranging from a correlation coefficient of *r_grayscale, edge_* = 0.470 (*p* < 0.01; 95% CI: 0.376–0.565) to *r_edge,thresholded_* = 0.736 (*p* < 0.01; 95%, CI: 0.671–0.801). Each of the synthetic image types also correlated moderately with the subcategory of abstract paintings (*r_edge,abstract_* = 0.334, *p* < 0.01; 95% CI: 0.229–0.438; *r*_thresholded,abstract_ = 0.368, *p* < 0.01; 95% CI: 0.264–0.472; *r_grayscale, abstract_* = 0.382, *p* < 0.01; 95% CI: 0.281–0.484). The correlations were lower for the preferences between different art subcategories with moderately strong correlations only between mountain and river paintings (*r_mountain, river_* = 0.355, *p* < 0.01; 95% CI: 0.256–0.453), and buildings and river paintings (*r_buildings, river_* = 0.388, *p* < 0.01; 95% CI: 0.283–0.493).

The overall correlation between preferences averaged across different synthetic images and averaged across different painting categories was significant (*r_synthetic,p aintings_* = 0.384, *p* < 0.01; 95% CI: 0.289–0.478). We also calculated this correlation for each individual participant with the corresponding frequency distribution and box plot graph depicted in the left and right panels of **Supplementary Figure 2**. The median of individual correlations was 0.596, with 37.4% of individual correlation coefficients above 0.8%.

#### Clustering Analysis

**FIGURE 4:**
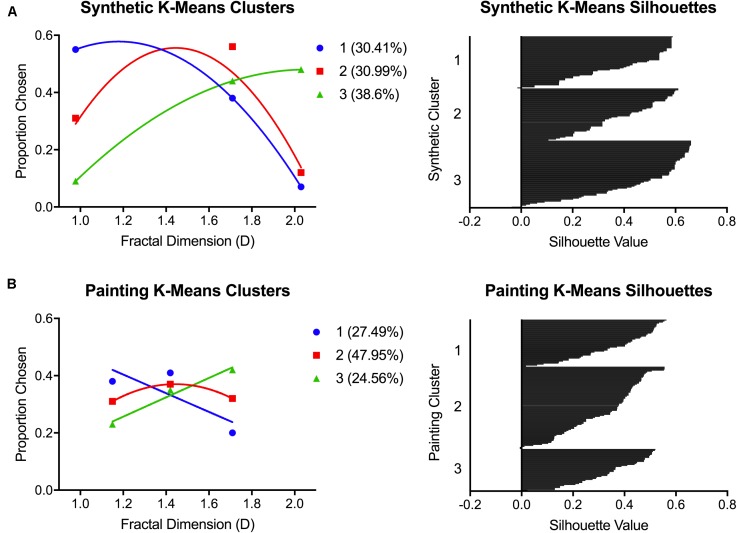
K-means clustering outcomes from Experiment 1. Graphs show the preference patterns characterizing each of the three K-means clusters, and the respective silhouette plots, for **(A)** synthetic images and **(B)** paintings.

To distinguish the main patterns of individual preferences, we conducted separate K-means clustering analyses for the average painting and synthetic fractal preferences. A three-cluster solution was chosen to evaluate whether the preference patterns observed would match those found in our previous study (Spehar et al., 2016). The average preferences for participants in each of the clusters, and the respective plots of silhouette values, for both synthetic fractal and painting images are depicted in **Figure 4**. Clusters and silhouette values were calculated using the K-means and silhouette functions in SPSS with a default squared Euclidian distance measure used. The silhouette values represent how similar a data point is to its own cluster (cohesion) compared to other clusters (separation). The silhouette values range from –1 to +1, with larger values implying that a data point is well matched to its own cluster. A large average silhouette value implies good clustering with the distances between data points in the same cluster minimized and the separation between different clusters maximized. The average silhouette values for the synthetic fractal and painting cluster solution were 0.431 (95% CI: 0.404–0.459) and 0.344 (95% CI: 0.322–0.366), respectively.

For the synthetic fractal images, K-means clustering revealed three distinct patterns of preference: (1) low D preference (30.41%), (2) intermediate D preference (30.99%), and (3) high D preference (38.60%). For the paintings, these preference patterns could also be distinguished: (1) low/intermediate D preferences (27.49%), (2) intermediate D preference (47.95%), and (3) high D preference (24.56%). However, in paintings, the clusters defined by low and intermediate preference patterns were less distinct in comparison to the synthetic fractal images. We conducted a correlational analysis to determine whether an individual’s membership to a particular cluster for synthetic stimuli was predictive of their painting cluster membership. We found a significant correlation of *r* = 0.272 [*F*_(1,170)_ = 13.536, *p* < 0.01] between synthetic fractal and painting cluster membership.

As an additional metric of internal consistency in preference between synthetic fractal and art images, we also calculated what proportion of subjects remained in the same cluster from synthetic to painting preference. We found that 68 of the 171 participants (39.8%) were consistent in their cluster membership and exhibited the same preference pattern across both stimulus types. For the remaining 103 participants, we examined the nature of the preference change between synthetic and art images. In sum, when the change happened, it almost always involved switching to an adjacent preference category. For example, 30 participants switched from liking intermediate synthetic images to preferring either high (*n* = 14) or low (*n* = 16) complexity paintings. Another 57 of participants switched from preferring either high (*n* = 36) or low (*n* = 21) synthetic images to preferring intermediate paintings. The preference changes in these majority cases were all toward adjacent image categories. In contrast, only 16 participants changed preferences from one extreme to the other: low D synthetic to high D painting preference (*n* = 7) and vice versa (*n* = 9).

#### Pleasantness, Interestingness, and Complexity Ratings

**FIGURE 5:**
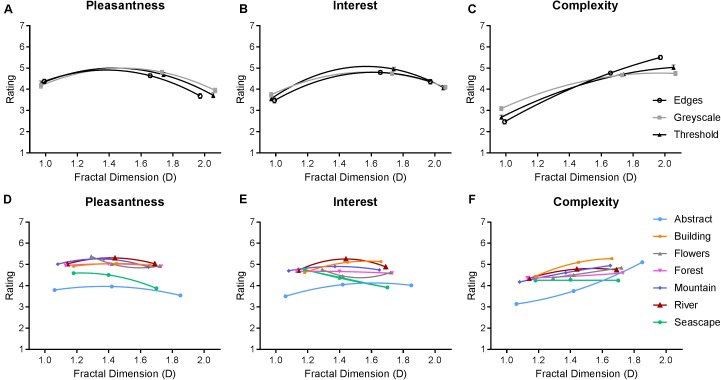
Average ratings from Experiment 1, where a higher value indicates greater pleasantness, interest, or complexity. Top row **(A–C)** depicts average (and SE) ratings for all three image categories of synthetic images. Bottom row **(D–F)** depicts average (and SE) ratings for all seven categories of paintings.

Average ratings of pleasantness, interestingness, and complexity are depicted in **Figure 5**. We performed two separate repeated-measures ANOVAs to analyze the effect of image category and fractal dimension within both classes of synthetic and art images, on all three rating scales. For synthetic images, multivariate analyses indicated a significant main effect of fractal dimension on ratings [*F*_(6,674)_ = 93.380, *p* < 0.01]. The main effect of image type (grayscale, thresholded, and edges) was not significant [*F*_(6,674)_ = 1.018, *p* = 0.412]. Averaged across image category, intermediate D images were rated as both more pleasant [*F*_(1.564,265.833)_ = 29.459, *p* < 0.01] and more interesting [*F*_(1.668,283.549)_ = 59.525, *p* < 0.01] compared to low D and high D images. Higher D images were also consistently rated as more complex than both low and intermediate D [*F*_(1.438,244.459)_ = 246.550, *p* < 0.01]. The interaction effect [*F*_(12,2040)_ = 15.999, *p* < 0.01] revealed that the relationship between ratings and fractal dimension varied depending on image category. Intermediate D edge images were rated as more pleasant than intermediate D grayscale images. High D edge images were also rated as more complex compared to high D grayscale and thresholded images.

For paintings, multivariate analyses indicated significant main effects of image category [*F*_(18,3050)_ = 37.765, *p* < 0.01] and fractal dimension [*F*_(6,674)_ = 36.940, *p* < 0.01]. Both abstract and sea paintings were given significantly lower average pleasantness and interest ratings compared to all other painting categories (*p* < 0.01), with the former also being rated even lower than the latter (*p* < 0.01). Similarly, abstract and sea paintings were rated as less complex compared to most other painting categories, and, in addition, the building paintings were rated as more complex compared to all other categories (*p* < 0.01).

Averaging over painting category, intermediate D paintings were given significantly higher ratings of pleasantness compared to high D paintings (*p* < 0.01) but not low D (*p* = 0.126). Intermediate D paintings were also rated as more interesting than both low D (*p* < 0.01) and high D (*p* = 0.01) paintings. Complexity ratings increased with increasing fractal dimensions-intermediate D paintings were rated as more complex than low D paintings (*p* < 0.01), and high D paintings were rated as more complex than both intermediate (*p* < 0.01) and low D (*p* < 0.01).

However, an interaction revealed that these outcomes depended on specific painting categories [*F*_(36,6110)_ = 14.876, *p* < 0.01]. Though the nature of these interactions is too numerous to fully outline, we can summarize that this interaction seems driven by both abstract and sea paintings being rated low in pleasantness, but this decreased rating was particularly large when the images had a high fractal dimension. On the other hand, flower paintings had a notably greater pleasantness rating compared to other painting categories, but this effect was most pronounced for low D images. Most painting categories had greater complexity ratings for higher D images; however, this increase was particularly steep for abstract images and followed a more curvilinear function for sea paintings.

#### Within-Subject Correlations Between 3AFC Preference and Complexity, Interestingness, and Pleasantness Ratings

In order to compare ratings of interest, complexity, and pleasantness for synthetic fractal and art images as a function of their respective fractal dimension (low, intermediate, and high), we calculated the mean of each rating averaged across the different categories in each image type (grayscale, thresholded, and edges and seven subcategories of paintings, respectively). We were interested in the intercorrelations between each rating, as well as in their correlation with 3AFC preference, both within and across the synthetic and art image type. This totalled 28 pairwise correlations calculated for each subject. The matrix of these correlations, averaged across all participants, can be found in **Supplementary Figure 3**.

Overall, ratings of interest and pleasantness closely correlated within their respective image types for both synthetic fractals (*r* = 0.529, *p* < 0.01; 95% CI: 0.433–0.625) and paintings (*r* = 0.454, *p* < 0.01; 95% CI: 0.358–0.551). Pleasantness and interest ratings for synthetic images were also good predictors of 3AFC synthetic preference (*r_pleasantness,preferenc_e* = 0.618, *p* < 0.0; 95% CI; 0.530–0.706 and *r_interest,preference_* = 0.508, *p* < 0.01; 95% CI: 0.412–0.604) and to a lesser degree, 3AFC painting preference (*r_pleasantness,preference_* = 0.316, *p* < 0.01; 95% CI: 0.216–0.416 and *r_interest,preference_* = 0.376, *p* < 0.01; 95% CI: 0.281–0.470). For both synthetic images and paintings, complexity was the weakest predictor of both preference and pleasantness, but had a low-to-moderate correlation with interest within the respective image categories (*r_complexity,interest_* = 0.478, *p* < 0.01; 95% CI: 0.381–0.576 for synthetic and *r_complexity,interest_* = 0.349, *p* < 0.01; 95% CI: 0.239–0.460 for paintings).

## Experiment 1: Discussion

Experiment 1 investigated the effect of fractal dimension on esthetic preferences across both synthetic fractal images and real-world artworks, and the consistency of these preferences within an individual. The study found that average preferences for both synthetic fractal images and paintings peaked for images in the intermediate D range. When analyzed by separate image categories, we found that the three variations of synthetic fractal images (grayscale, thresholded, and edges) all separately exhibited preference peaks at the intermediate D level. The same was true for most painting categories, with the exception of flowers and forests whereby low D paintings were most preferred. These findings support a long line of studies that have observed the same preference peak in images characterized by intermediate fractal-scaling statistics (Aks and Sprott, 1996; Spehar et al., 2003, 2015; Hagerhall et al., 2004; Street et al., 2016).

Furthermore, the above findings were complemented with the overall ratings of interestingness, complexity, and pleasantness for both synthetic fractal and art images. Both perceived pleasantness and interestingness peaked for images in the intermediate D group were significant predictors of 3AFC preference for art and, in particular, for the synthetic fractal images. Complexity ratings were significantly higher in image groups characterized by greater fractal dimensions, reaffirming the role of D as a reliable measure of complexity.

We were also interested in the extent to which individual participants would exhibit the same preference for certain fractal-scaling characteristics across the different subcategories of synthetic and art images and between synthetic and art images overall. We found that the within-subject agreement in preference between the three different types of synthetic patterns was remarkably high and closely paralleled the pairwise within-observer correlations observed previously in these types of synthetic images (Spehar et al., 2016). While the within-subject agreement in preference between different painting subcategories was slightly lower, the overall correlation between synthetic images (averaged across different synthetic image categories) and art (averaged across different painting categories) was moderately high, with a mean of *r_synthetic,painting_* = 0.384 (*p* < 0.01) and a median of *r* = 0.596. In fact, in our sample of 171 participants, 37.4% correlation coefficients were above *r* = 0.8 and 59.6% were of above *r* = 0.4.

We performed clustering analyses to further characterize the nature of individual variations in preference for fractal-scaling characteristics. This revealed three main preference patterns that replicate those described in Spehar et al. (2016). For both synthetic fractal images and paintings, the clusters clearly distinguished individual observers into groups who preferred either high-, intermediate-, or low-complexity images. The correlation between synthetic and painting cluster membership was significant and positive but relatively modest. Further analysis indicated that 39.8% of participants demonstrated exactly the same preference patterns across the two image categories. However, when there was a change in preference clusters between synthetic and art images, the vast majority of subjects (50.8%) either transferred from an intermediate D preference to either high or low, or regressed back from the extremes to intermediate. In contrast, only 9.4% of participants shifted their preferences from one extreme to another. This finding is promising as it suggests that complexity preferences, while slightly fluid, do have a certain level of stability and typically will not change drastically from one extreme to another.

While the above analyses suggest a promising link between preference for fractal scaling in synthetic and art images, we believe that the strength of this relationship might have been underestimated by our decision to use a wider range of fractal dimension variations in the synthetic images compared to that of the paintings. The range of fractal-scaling variations chosen for synthetic images in this study was the same as those used in our previous laboratory-based studies (Spehar et al., 2015, 2016). Doing this allowed us to make direct comparisons between the results of these past studies and the results of the present one, which were obtained through a novel, and possibly less-controlled, online procedure. Primarily, we chose this wider range to ensure that the manipulation of fractal dimension was robust despite the different experimental contexts, and to have the opportunity to adjust our experimental design in case it was not. Consequently, the synthetic images in our low fractal dimension category averaged at approximately D_synthetic_ = 0.98 compared to *D*_paintings_ = 1.15 for paintings. Similarly, the D values for intermediate (*D*_synthetic_ = 1.71 compared to *D*_paintings_ = 1.42) and high (*D*_synthetic_ = 2.03 compared to *D*_paintings_ = 1.71) dimension categories also differed between synthetic and painting image types. Furthermore, the average fractal dimension of the intermediate D synthetic stimuli was a lot closer to the average D value of the high D paintings than the intermediate D paintings. It is possible that 3AFC preference was more sensitive to these differences than expected, and individuals who exhibited one preference pattern for synthetic stimuli may not have reflected the same pattern for paintings.

To investigate the possibility that the range of dimensions used for our synthetic fractal stimuli may have affected preference consistency, in Experiment 2, we reduced the range of D values to more closely match those of our paintings. We predicted that this change in range will increase the within-subject correspondence in synthetic image and painting preferences.

## Experiment 2: Materials and Methods

### Design

The same 10 × 3 within-subjects design described in Experiment 1 was adopted here. Image type consisted of 10 categories: 3 types of synthetic fractal images (grayscale, edges, and thresholded) and 7 types of paintings (abstract, buildings, flowers, forests, mountains, rivers, and seas). All image categories varied on three levels of fractal dimension: low, intermediate, and high. However, the fractal dimension of our synthetic stimuli now matched the average D values of the paintings in each of the low, intermediate, and high D groups. Four outcomes were measured: preference, pleasantness, complexity, and interest.

### Participants

A total of 181 participants were recruited via the online recruitment platform MTurk^1^. Two subjects failed to complete the task and were excluded from the final analysis. Of the remaining 179 subjects, 42% were female and the mean age was 34.06 years. A total of 84% indicated their country of residence as the United States, 8% as India, and 6% as the United Kingdom. Participants underwent the same consent procedure as described in Experiment 1 and were reimbursed with US$4.50 on the study’s completion.

### Materials and Apparatus

#### Synthetic Fractal Images

**FIGURE 6:**
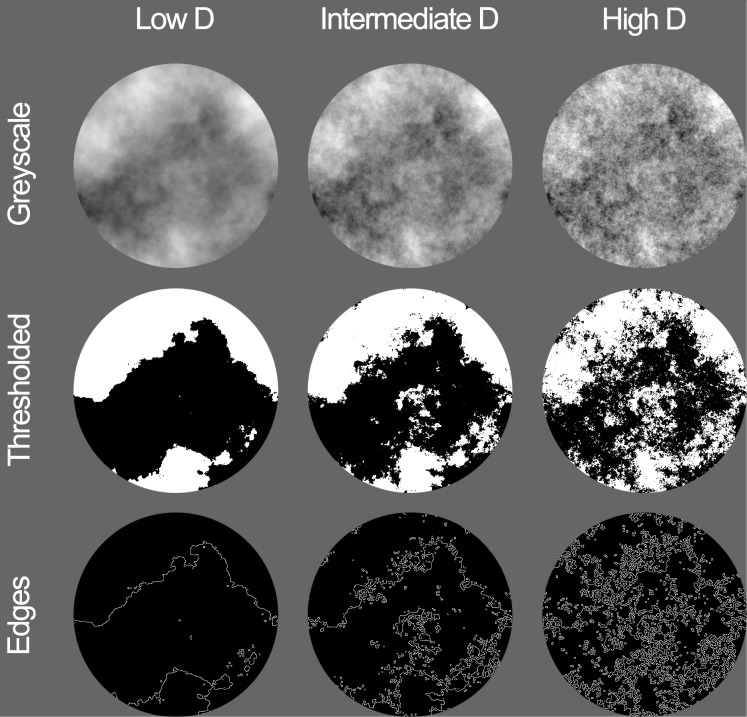
Synthetic fractal stimuli used for Experiment 2. Image types shown from top to bottom are grayscale, thresholded, and edges with the average fractal dimension D of 1.209 (left), 1.491 (middle), and 1.819 (right), respectively.

Synthetic images were generated using the method described in Experiment 1 except using three different levels of amplitude spectrum slope, α = 1.1, 1.4, and 1.7. These slopes corresponded to averaged measured fractal dimensions of *D* = 1.74, 1.42, and 1.13, respectively (see **Table 2**). Edge and thresholded variations of the same grayscale noise patterns were also created, resulting in 36 (4 seeds × 3 categories × 3 fractal dimensions) total fractal noise images (**Figure 6**).

**Table 2:** Average fractal dimension and luminance values for each category of paintings in Experiment 2.

	Fractal dimension			Luminance		
	Low	Intermediate	High	Low	Intermediate	High
Grayscale	1.10	1.39	1.74	127.5	127.5	127.5
Thresholded	1.10	1.39	1.74	129.0	127.4	127.2
Edges	1.20	1.48	1.74	5.025	17.28	37.37
Mean	1.13	1.423	1.74	87.16	90.72	97.37

#### Paintings

The same set of paintings from Experiment 1 was used.

### Procedure

With the exception of the synthetic fractal images having a narrower D range, the procedure and analyses remained the same as described in Experiment 1.

## Experiment 2: Results and Discussion

### 3AFC Preference Task

#### Population Preferences for Images With Low, Intermediate, and High Fractal Dimension

**FIGURE 7:**
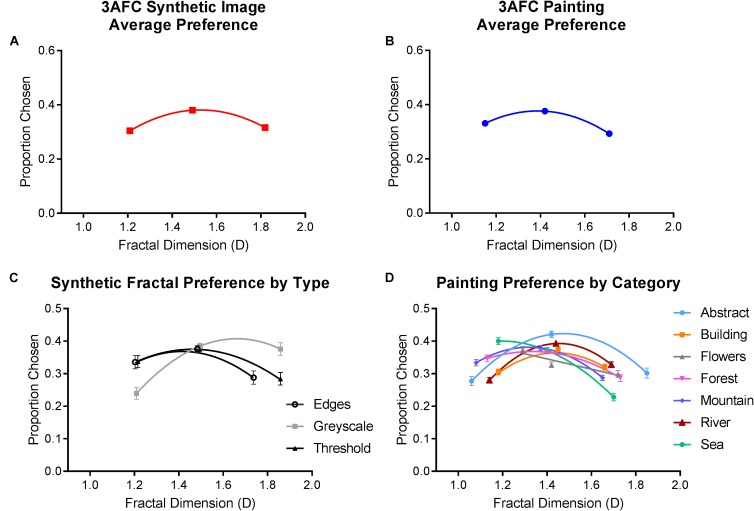
Average preferences from Experiment 2. Top row depicts the average (and SE) proportion of times **(A)** synthetic and **(B)** painting from each level of fractal dimension were selected. Bottom row shows the average (and SE) proportion of times each category within the **(C)** synthetic and **(D)** painting image types was chosen, plotted as a function of their measured fractal dimension.

The average preference for synthetic images and paintings, and the breakdown within each category, are shown in **Figure 7**. Repeated-measures ANOVAs were performed separately for synthetic image and painting preferences. Fractal dimension and image category were set as independent variables.

For synthetic images, there was a significant effect of fractal dimension [*F*_(1.336,237.750)_ = 5.960, *p* = 0.009] and a non-significant effect of image category [*F*_(1.439,256.123)_ = 3.148, *p* = 0.061]. Intermediate D images made up 38% of choices, which was significantly higher than the proportion of times low D images (30.4%, *p* < 0.01) and high D images (31.6%, *p* = 0.005) were chosen. A significant interaction between image category and fractal dimension indicated that preference for high and low fractal dimensions differed significantly for grayscale images compared to edge and thresholded ones [*F*_(2.682,477.371)_ = 12.481, *p* < 0.01]. The proportion of times low D grayscale images were chosen was significantly lower than the thresholded and edge images, and the proportion of times high D grayscale images were chosen was significantly higher.

Similarly for paintings, there were significant effects of fractal dimension [*F*_(1.459,259.742)_ = 33.964, *p* < 0.01] and painting category [*F*_(5.733,1020.441)_ = 3, *p* = 0.007]. Intermediate D paintings were chosen 37.6% of the time, which was significantly more than both low (33.1%, *p* < 0.01) and high D (29.3%, *p* < 0.01). As in Experiment 1, preference patterns across the various painting categories also varied slightly. Almost all categories exhibited a greater preference at the intermediate D level. However, low D preferences were the greatest for flower and sea paintings.

#### Internal Consistency in Preference for Different Fractal Dimensions Across Image Types

As described in Experiment 1, we conducted a correlational analysis to determine the consistency of individual preferences across synthetic fractal images and paintings. Overall, the correlations in 3AFC preference between synthetic fractal and art images, as well as the correlations between their respective image categories, closely matched those found in Experiment 1 (**Supplementary Figure 4**). The average correlation between overall synthetic image and painting preferences was *r* = 0.369, *p* < 0.01; 95% CI: 0.273–0.465, with 44.07% of participants exhibiting preference correlations of 0.8 and above, and 57.06% of participants exhibiting correlations of 0.6 and above (**Supplementary Figure 5**). Synthetic fractal image subcategories were moderately correlated within themselves (*r_grayscale,edge_* = 0.324, *p* < 0.01; 95% CI: 0.210–0.438 to *r_threshold, edge_* = 0.592, *p* < 0.01; 95% CI: 0.498–0.687) and also with abstract art preferences (*r_abstract, synthetic_* = 0.358, *p* < 0.01; 95% CI: 0.256–0.460). With the exception of grayscale, both edge and thresholded images had overall low, positive correlations with different categories of paintings. Similarly, correlations between painting category preferences were positive, but not as strongly correlated as synthetic fractal image categories.

#### Clustering Analysis

**FIGURE 8:**
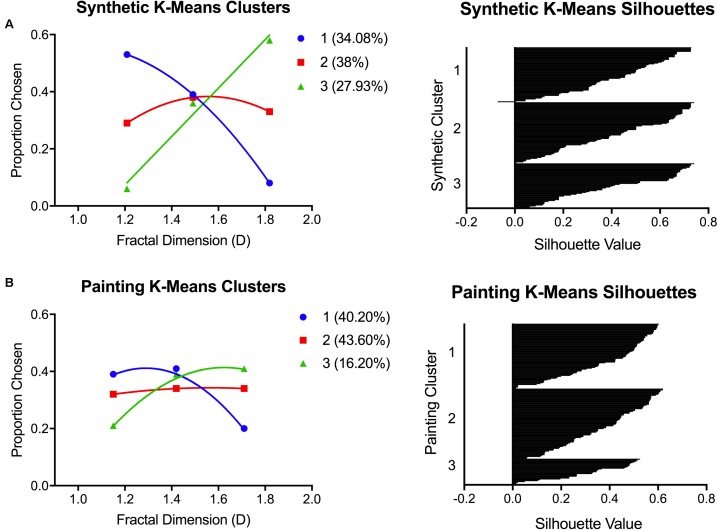
K-means clustering outcomes from Experiment 2. Graphs show the preference patterns characterizing each of the three K-means clusters, and the respective silhouette plots, for **(A)** synthetic images and **(B)** paintings.

We conducted separate K-means clustering analyses for painting and synthetic fractal preferences in SPSS using the three-cluster solution motivated by both our previous findings (Spehar et al., 2016) and the results of Experiment 1 (**Figure 8**).

For synthetic images, the three clusters were uniquely distinguished by subjects who preferred: (1) low D (34.08%), (2) intermediate D (38%), and (3) high D (27.93%). For paintings, the clusters were distinguished as those who preferred: (1) low/intermediate D (40.20%), (2) intermediate D (43.60%), and (3) high D (16.20%). Similar to Experiment 1, the different preference patterns for paintings were less distinct compared to those for synthetic images. The low D preference pattern for paintings can be interpreted as a preference for low/intermediate images rather than a clear low D preference. Despite this, we again found a significant correlation (*r* = 0.404, *p* < 0.01) between synthetic and painting preference clusters. This was higher than the correlation found between clusters in Experiment 1 (*r* = 0.272).

Finally, we calculated the proportion of subjects who remained in the same preference cluster across the two image types. We found that 92 of the 179 (51.44%) subjects who were members of a particular preference cluster for synthetic images were members of the same preference cluster for paintings. The remaining 87 subjects all changed cluster membership between the two images types. To break it down, 32 subjects who preferred intermediate D synthetic images preferred either high (*n* = 7) or low (*n* = 25) D paintings. In total, 42 of subjects who preferred either high (*n* = 23) or low (*n* = 19) synthetic images preferred intermediate D paintings. The final 13 subjects consisted of those who either preferred low D synthetic and high D paintings (*n* = 4), or high D synthetic and low D paintings (*n* = 9), that is, their preference shifted from one extreme of D to the other.

#### Pleasantness, Interestingness, and Complexity Ratings

**FIGURE 9:**
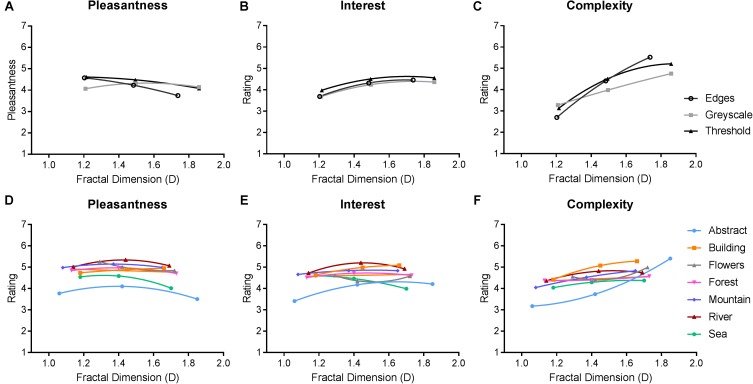
Average ratings from Experiment 2. Top row **(A–C)** depicts average (and SE) ratings for all three image categories of synthetic images. Bottom row **(D–F)** depicts average (and SE) ratings for all seven categories of paintings.

Average ratings of pleasantness, interestingness, and complexity are displayed in **Figure 9**. Two separate multivariate, repeated-measures ANOVAs analyzed the effect of image category and fractal dimension on the three rating measures: pleasantness, complexity, and interest.

For synthetic images, there was a main effect of image type [*F*_(6,706)_ = 3.344, *p* < 0.01] in which thresholded images were rated as more pleasant and interesting compared to both grayscale and edge variations, and more complex compared to grayscale synthetic fractal images. The main effect of fractal dimension was significant [*F*_(6,706)_ = 103.437, *p* < 0.01] with higher ratings of pleasantness given to low D images. Interestingly, the reverse trend was found for interest ratings, which was the lowest for low D images and increased significantly for intermediate and high D. Finally, high D images were rated as more complex compared to both intermediate (*p* < 0.01) and low D images (*p* < 0.01). Results also indicated a significant interaction effect between image category × fractal dimension for pleasantness [*F*_(3.205, 570.510)_ = 18.013, *p* < 0.01] and complexity ratings [*F*_(3.650,649.725)_ = 44.768, *p* < 0.01]. Low D grayscale images were given lower pleasantness ratings compared to low D thresholded and edge images. The direction of effect between these image types reversed for intermediate and high D images, but not to significant effect. In regard to complexity, low D edge images were rated as less complex than both grayscale and thresholded variations in the same low D group. However, this is reversed in the high D group where edge images were rated as more complex than the grayscale and thresholded variations.

For paintings, all ratings varied as a function of painting category [*F*_(18,3194)_ = 33.925, *p* < 0.01] and fractal dimension [*F*_(6,706)_ = 53.613, *p* < 0.01]. Ratings for paintings were similar to those found in Experiment 1. Both abstract and sea paintings were given the lowest ratings of pleasantness and interest (*p* < 0.01) compared to all other painting categories, and building paintings were rated as the most complex. Pleasantness ratings differed significantly across fractal dimension [*F*_(1.688,300.402)_ = 19.484, *p* < 0.01]. Averaging across painting categories, results showed that intermediate D paintings were rated as more pleasant than both high D (*p* < 0.01) and low D paintings (*p* = 0.01). Similarly, intermediate D paintings were considered significantly more interesting than low D (*p* < 0.01), though not high D (*p* = 0.270) paintings. Intermediate D paintings were rated as more complex than low D paintings (*p* < 0.01), and high D paintings were rated as more complex than intermediate (*p* < 0.01) and low D (*p* < 0.01) paintings. A significant interaction [*F*_(36,6398)_ = 18.218, *p* < 0.01] revealed similar outcomes for painting ratings to those found in Experiment 1.

#### Within-Subject Correlations Between 3AFC Preference and Complexity, Interestingness, and Pleasantness Ratings

We calculated the correlation between a subject’s scores on one rating scale with their scores on all other rating scales, as well as their 3AFC preferences (**Supplementary Figure 6**). Ratings of pleasantness for both painting and synthetic fractal images were significant predictors of 3AFC preference for paintings (*r* = 0.409, *p* < 0.01; 95% CI: 0.311–0.507 and *r* = 0.303, *p* < 0.01; 95% CI: 0.200–0.407, respectively). Interest was closely correlated with the ratings of pleasantness for both paintings (*r_interest,pleasantness_* = 0.509, *p* < 0.01; 95% CI: 0.419–0.600) and synthetic fractal images (*r_interest,pleasantness_* = 0.365, *p* < 0.01; 95% CI: 0.259–0.472). In addition, interest was also a significant predictor of 3AFC preference for both paintings (*r_interest,preference_* = 0.302 *p* < 0.01; 95% CI: 0.201–0.404) and synthetic image (*r_interest,preference_* = 0.367, *p* < 0.01; 95% CI: 0.259–0.475). Complexity ratings for synthetic fractal images and paintings were closely correlated with each other (*r_synthetic,painting_* = 0.601, *p* < 0.01; 95% CI: 0.516–0.686). Furthermore, ratings of complexity also moderately correlated with interest for both paintings (*r_interest,complexity_* = 0.345, *p* < 0.01; 95% CI: 0.239–0.450) and for synthetic fractal images (*r_interest,complexity_* = 0.369, *p* < 0.01; 95% CI: 0.253–0.486).

#### Comparison of Results Between Experiment 1 and Experiment 2

In Experiment 2, we manipulated the average fractal dimension of the low, intermediate, and high D groups so that they more closely matched those of the paintings. Comparing the two experiments, we expected that preference and rating patterns would not significantly differ for paintings, but may to some degree for synthetic images. We combined the data sets (averaged over image category) from both experiments and performed a multivariate, repeated-measures ANOVA, indicating experiment as the between-subjects variable, and fractal dimension and image type as within-subjects variables. The measures compared included preference, pleasantness, complexity, and interest.

**FIGURE 10:**
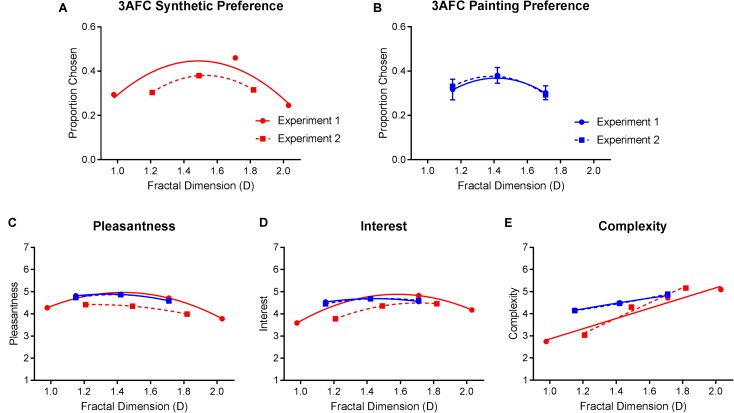
Top row compares average (and SE) preference for **(A)** synthetic images and **(B)** paintings, across Experiments 1 and 2. Bottom graphs compare average (and SE) ratings of **(C)** pleasantness, **(D)** interest, and **(E)** complexity for both synthetic and painting images between the two experiments. All measures are plotted as a function of the stimuli’s fractal dimension values.

Univariate comparisons of individual measures between experiments, averaged over fractal dimension and stimulus type, indicated no significant differences in ratings of pleasantness [*F*_(1,348)_ = 0.081, *p* = 0.777], complexity [*F*_(1,348)_ = 0.019, *p* = 0.891], and interest [*F*_(1,348)_ = 0.013, *p* = 0.910]. However, analyses did reveal a significant fractal dimension × experiment × image type interaction for preference [*F*_(1.57,546.252)_ = 12.560, *p* < 0.01], pleasantness, [*F*_(1.494,519.981)_ = 8.999, *p* = 0.001], complexity [*F*_(1.598,556.167)_ = 11.509, *p* < 0.01], and interest [*F*_(1.644,572.017)_ = 16.144, *p* < 0.01]. This interaction essentially indicated that the differences between synthetic images on these measures, between Experiments 1 and 2, were greater than they were for paintings. For example, there were no significant differences in painting preference between Experiments 1 and 2 across any values of fractal dimension. However, the average synthetic image preference in Experiment 2 was significantly lower at intermediate D and significantly higher at high D compared to Experiment 1 (**Figure 10**).

Congruent with our prediction, within-subject agreement in preference patterns between synthetic fractal images and paintings was greater when the range of fractal-scaling variations between them were more closely matched. For example, compared to Experiment 1 in which 39.8% of participants were members of the same preference cluster for synthetic images and paintings, 51.44% were members of the same preference clusters in Experiment 2. Similarly, while 29.2% of subjects shifted between intermediate and high D preferences in Experiment 1, Experiment 2 found fewer subjects (16.8%) shifted between these two preference patterns.

## General Discussion

Across both experiments, average preference for paintings and synthetic images peaked for intermediate D. These results were congruent with the well-established inverted U-curve preference–complexity relationship first proposed by Berlyne (1971) and with a number of more recent studies (Spehar et al., 2015; Güçlütürk et al., 2016). Furthermore, an analysis of individual differences highlighted three distinctive preference patterns (percentages reported are averaged values across both stimulus types and experiments): preference for low fractal dimension (33.05%), intermediate fractal dimension (40.13%), and high fractal dimension (26.82%). These preference patterns were consistent across both synthetic fractal images and artworks. Furthermore, the consistency in preference between the two image types was improved by the closer matching of fractal dimension between paintings and synthetic images in Experiment 2. While average preference correlations between paintings and synthetic images were not notably different between the two experiments (*r* = 0.384 in Experiment 1, *r* = 0.366 in Experiment 2), the correlation between preference cluster membership was markedly higher in Experiment 2 (*r* = 0.272 in Experiment 1, *r* = 0.404 in Experiment 2).

This was likely because more subjects remained in the same preference cluster across stimulus type in Experiment 2 (51.40%), compared to Experiment 1 (39.77%). In both studies, it was found that preference patterns between synthetic and art images rarely changed from one extreme to another. In other words, subjects who preferred low D synthetic images less likely preferred high D paintings, and generally chose paintings that were congruent with a low-intermediate preference cluster (and vice versa). Taken together, these results reaffirm the same patterns of individual differences found in previous studies (Spehar et al., 2016) and broaden the predictive validity of considering fractal-scaling properties in esthetic preference beyond just artificially generated stimuli and into real-world artworks.

Average ratings of pleasantness, complexity, and interest for paintings were consistent across Experiments 1 and 2. Perceived pleasantness and interest peaked for intermediate D paintings and complexity ratings increased with greater D paintings. However, ratings differed between the two experiments for synthetic images. In Experiment 2, low D synthetic images were rated as the most pleasant and high D images were rated as the most interesting (as opposed to intermediate D for both measures in Experiment 1). Initially, these outcomes appear to dispute the patterns found in Experiment 1; however, this is not the case. When we map these average ratings as a function of their actual numerical D values along the same axis (**Figure 10**), we find that the shape of the function defined by the narrower D range in Experiment 2 overlaps almost exactly with the function defined by the wider range in Experiment 1. This further supports the idea that esthetic judgments were exquisitely sensitive to small variations regarding the underlying fractal-scaling properties of our stimuli (Spehar et al., 2003).

However, while the relationship between synthetic and art image preference is promising, it is important to address why certain categories of artworks simply did not show as high a correspondence with average preference patterns as others. Across both experiments, low D flower paintings were consistently preferred over both intermediate and high D options. The same preference for low D images is observed for forests, exclusively in Experiment 1, and for seascapes, exclusively in Experiment 2. As a result, these three categories of paintings also exhibited the lowest average preference correlations within individuals across both studies.

Interestingly, for these categories, paintings allocated to the higher D groups were not always regarded as more complex than lower D images. Across the two experiments, both low and high D flowers were rated as more interesting than intermediate D. The same effect was also observed for complexity in Experiment 1, though it was not replicated in Experiment 2. While these rating differences were not significant, they do highlight a potential reason for the discrepant preference patterns found in flower paintings. Flowers had a narrower range in D (1.290–1.718) due to values in the low D category being higher than the average for low D paintings. This resulted in flowers having the highest average fractal dimension of all paintings. Subsequently, the greater preference for low D images created by this reduced range in D would fit in line with our findings comparing the changes in range between Experiments 1 and 2. In the end, it was not surprising that low D flower paintings, with the average D value of 1.290, were the most preferred as this value falls well within the typical “highly pleasant” D range observed in previous preference studies (Spehar et al., 2003, 2016). Simply put, the fractal dimension of the low D flower paintings may not have been low enough to achieve the predicted effect.

These general discrepancies highlight the intrinsic problem to using stimuli like artworks in empirical studies. While fractal dimension offers a highly efficient and objective means of measuring structural complexity, it does not account for all aspects of variation in an image. While the shorter fractal dimension range may explain some of the unexpected outcomes in the average preference for flower paintings, it is certainly not the whole story. Future research into the specific effects of image properties such as luminance and its interaction with fractal dimension is strongly encouraged to provide clarity for our current findings.

We are also aware of the possible concerns regarding the online nature of our study, specifically of how this limits our control over subjects’ viewing conditions. Our previous studies have been conducted in controlled laboratory settings where we found the robust effect of fractal-scaling statistics on esthetic preference (Spehar et al., 2003, 2015, 2016). Although the variability in viewing conditions and display calibration could have potentially only served to weaken our effect sizes, we found that both population preferences and patterns of individual differences replicated the previous patterns with synthetic fractal noise images quite closely. We believe that these findings strengthen the generalizability of fractal-scaling influences in esthetic preference to variable viewing conditions.

Another possible factor that could have influenced our results is that some of the images might have been perceived to be of poorer photographic quality and thus rated low. For example, lowering the quality of photographs of natural scenes has been previously shown to decreased how much they were preferred (Tinio and Leder, 2009). Indeed, synthetic grayscale images with low D are characterized by a high degree of image blur and have been rated lower in esthetic appeal and pleasantness compared to the thresholded and edge images with the same low D values in current as well as in our previous studies (Spehar et al., 2015, 2016). These findings are consistent with a number of other studies that have shown the role of image blur in visual discomfort (Fernandez and Wilkins, 2008; Juricevic et al., 2010; O’Hare et al., 2013).

However, while image blur certainly seems to contribute to the observed low visual preference of grayscale images with low fractal-scaling properties, our synthetic edge and thresholded variants do not have compromised image quality. Yet thresholded and edge images with low fractal-scaling characteristics are still associated with lower visual preference compared to their counterparts with intermediate fractal-scaling characteristics. One can argue that with these images fractal scaling plays a more direct role in influencing perceived complexity and esthetic preference. Nevertheless, it would seem valuable to include a measure of perceived image quality in future studies of esthetic preference.

To conclude, this study advances the growing body of research implicating the integral role of fractal-scaling properties in empirical esthetics. Previously, studies elucidating the relationship between fractal scaling and esthetics in art have predominantly remained descriptive – highlighting the remarkably close overlap in statistical properties between artworks and natural scenes (Taylor et al., 1999; Redies et al., 2007b; Mather, 2014). Our study attempted to extend this framework to account for the different patterns of preference that emerge in art. Overall, our findings further support the role of fractal-scaling statistics as both a key determinant of an object’s esthetic value and as a valuable predictor of individual differences in esthetic preference across multiple contexts. Our findings also support the notion that the esthetic appeal of images possessing intermediate fractal-like statistics is highly robust but not universal. Distinctive patterns of preferences for different levels of fractal scaling can be easily discerned, and remain both inter- and intraindividually consistent across both synthetic images and art.

## Ethics Statement

Human participants were presented with an information statement prior to beginning the study outlining what the experiment involved and their rights to privacy and confidentiality. A debrief statement containing the aims and hypotheses of the study, the contact information of the investigators and relevant mental health helplines were included at the end of the study. Experimental procedures were approved by the UNSW Human Research Ethics Advisory Panel (HREAP-C).

## Author Contributions

BS and CV designed the study, analyzed, interpreted the data, and wrote the manuscript. CV ran the study, and collected and analyzed the data.

## Conflict of Interest Statement

The authors declare that the research was conducted in the absence of any commercial or financial relationships that could be construed as a potential conflict of interest.

## Footnotes

1 https://www.mturk.com/
