# Supplementary material for: Preference for Fractal-Scaling Properties Across Synthetic Noise Images and Artworks
Source: Front Psychol. 2018 Aug 29;9:1439. doi: 10.3389/fpsyg.2018.01439 (PMC6123544; doi:10.3389/fpsyg.2018.01439)
Supplement: Supplementary file 1 [file Data_Sheet_1.pdf]

Supplementary Material

Preference for fractal-scaling properties across synthetic noise images and artworks

Catherine Viengkham, Branka Spehar*

*** Correspondence:** Branka Spehar: b.spehar@unsw.edu.au

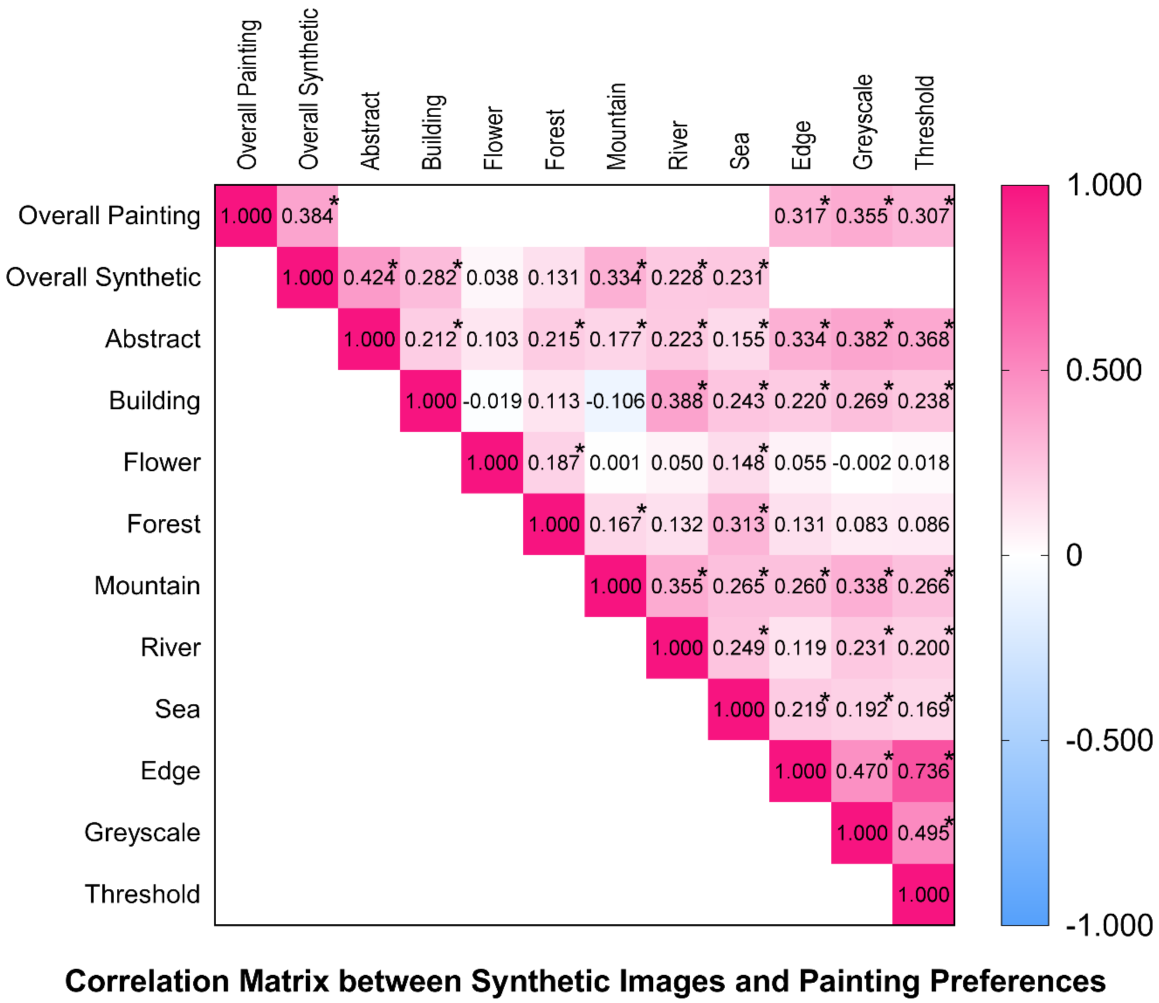

Supplementary Figure 1. A heat map of the average pairwise within-observer rank correlations for 3AFC preference between different image types and subcategories in Experiment (* $p < .0$). The correlations between the preference for the individual painting and synthetic image subcategories and overall painting and the overall synthetic preference (averaged across the individual painting and synthetic image subcategories) were not included.

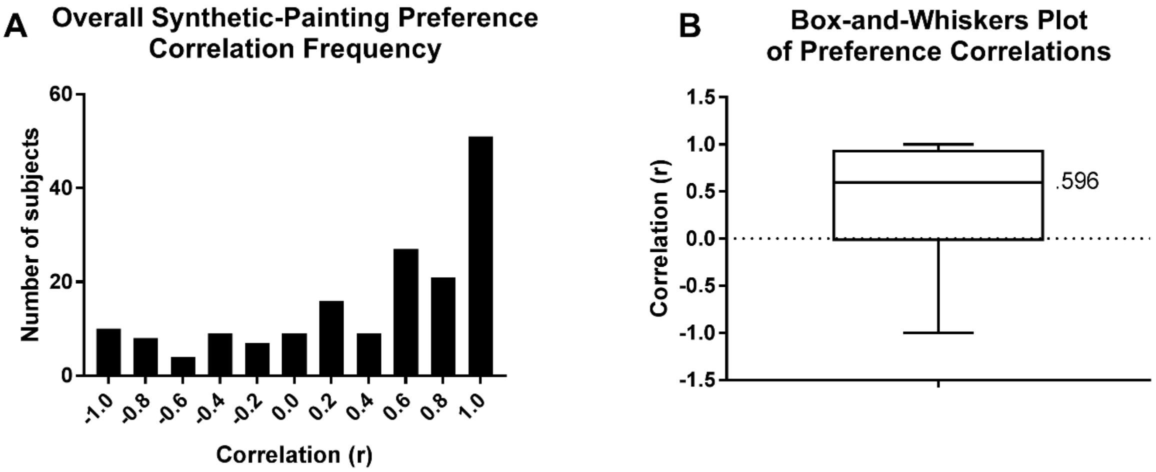

Supplementary Figure 2. Pairwise correlations between overall synthetic fractal and overall painting images in Experiment 1. (A) Histogram of the frequency distribution of pairwise synthetic-painting preference correlations for 171 subjects. (B) Boxplot of the same distribution. Mean correlation is .384 and median is .596.

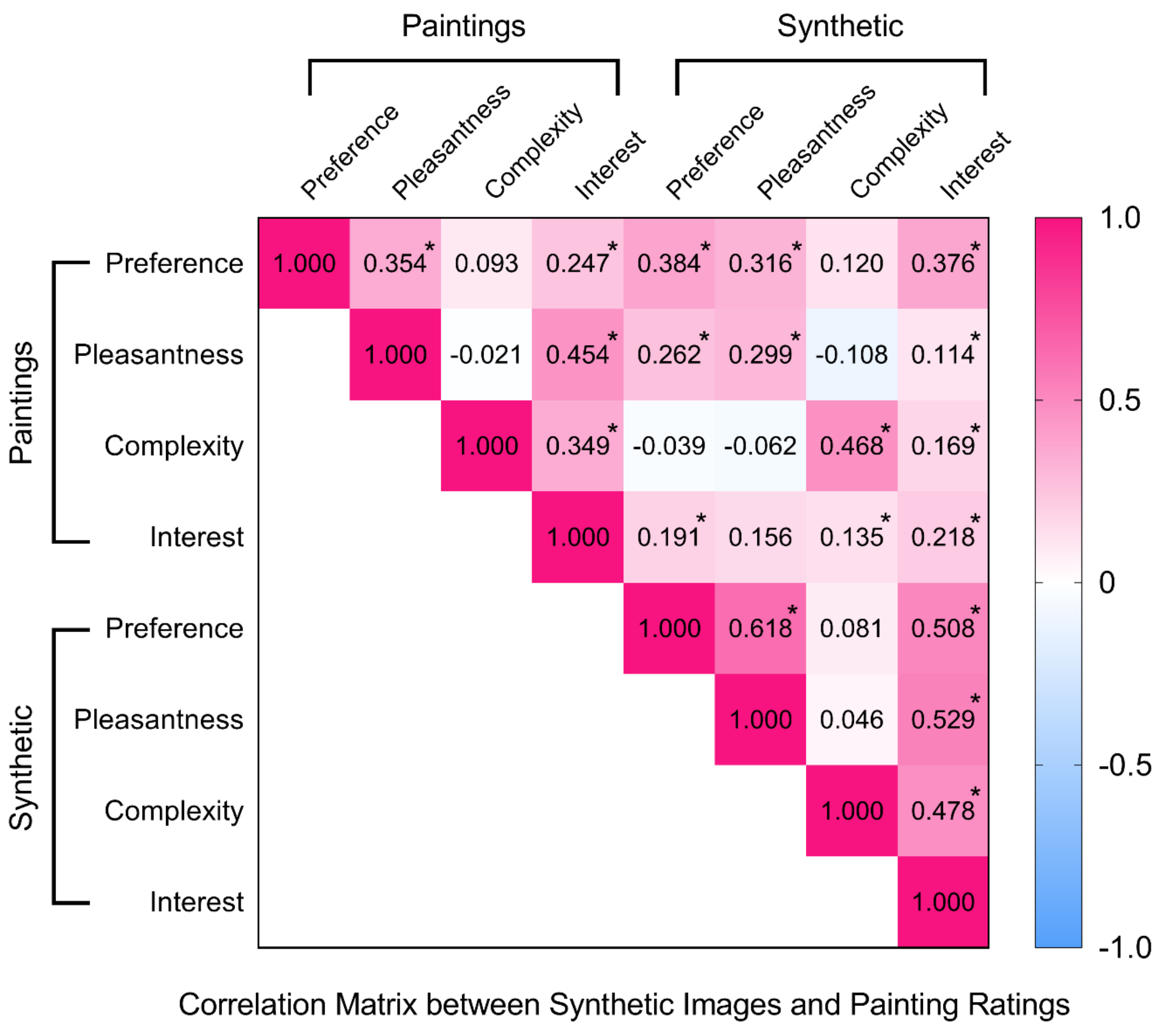

Supplementary Figure 3. A heatmap of the average pairwise correlation matrix between 3AFC and ratings of Complexity, Pleasantness and Interestingness in Experiment 1 (* $p < .01$).

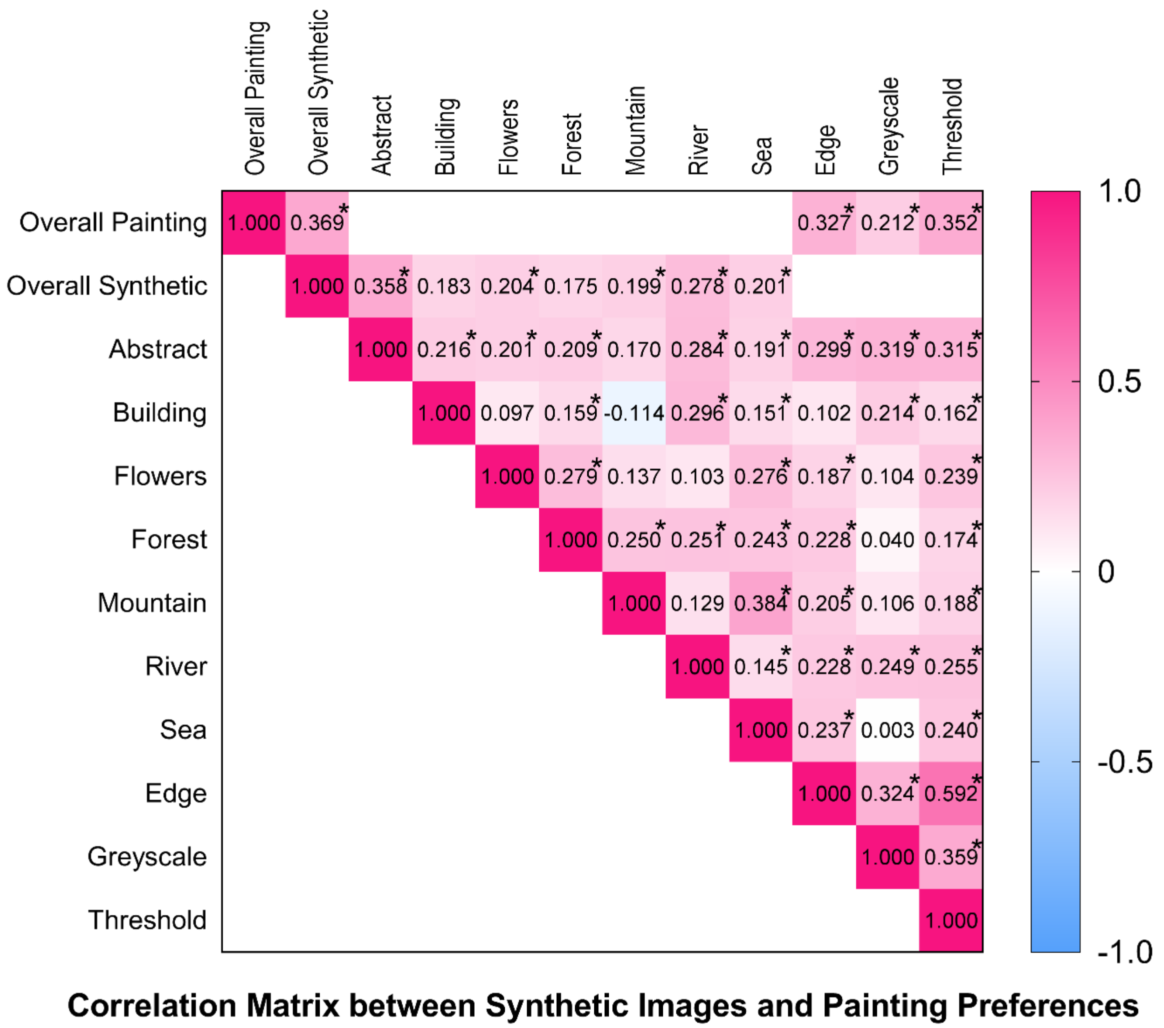

Supplementary Figure 4. A heat map of the average pairwise within-observer rank correlations for 3AFC preference between different image types and subcategories in Experiment 2 (* $p < .01$). The correlations between the preference for the individual painting and synthetic image subcategories and overall painting and the overall synthetic preference (averaged across the individual painting and synthetic image subcategories) were not included.

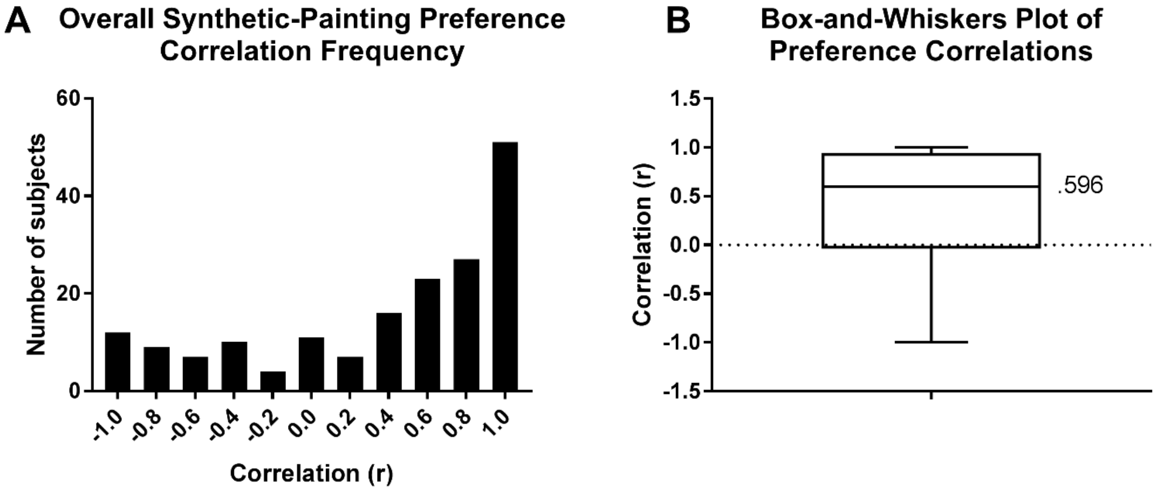

Supplementary Figure 5. Pairwise correlations between overall synthetic fractal and overall painting images in Experiment 2. (A) Histogram of the frequency distribution of pairwise synthetic-painting preference correlations for 179 subjects. (B) Boxplot of the same distribution. Mean correlation is .369 and median is .596.

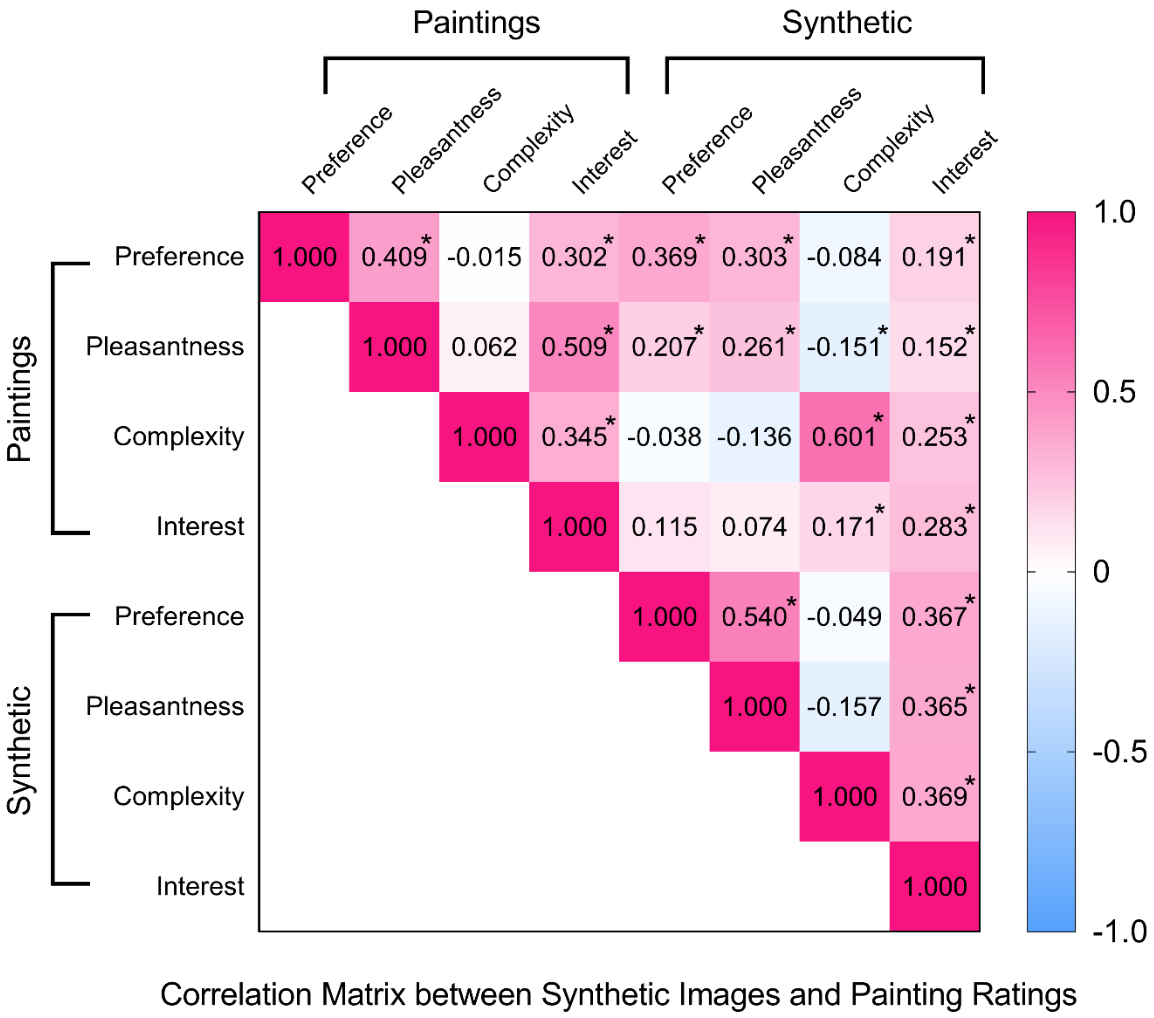

Supplementary Figure 6. A heatmap of the average pairwise correlation matrix between 3AFC and ratings of Complexity, Pleasantness and Interestingness in Experiment 2 (* p < .01).
