# Supplementary material for: Preference for Fractal-Scaling Properties Across Synthetic Noise Images and Artworks
Source: Front Psychol. 2018 Aug 29;9:1439. doi: 10.3389/fpsyg.2018.01439 (PMC6123544; doi:10.3389/fpsyg.2018.01439)
Supplement: Supplementary file 2 [file Presentation_1.pdf]

Supplementary Images

Preference for fractal-scaling properties across synthetic noise images and artworks

Catherine Viengkham, Branka Spehar*

*** Correspondence:** Branka Spehar: b.spehar@unsw.edu.au

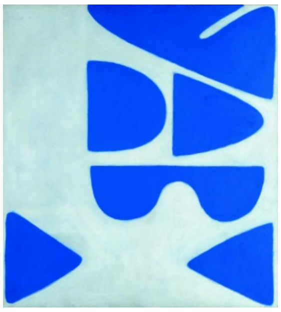

William Scott, Berline Blues 6, 1966

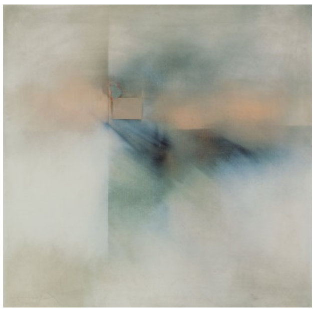

Fernado Zóbel, Flight in Pink, 1966

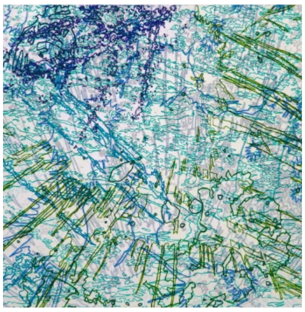

Ingrid Calame, Untitled (Trace 1, 2, & 3), 2006

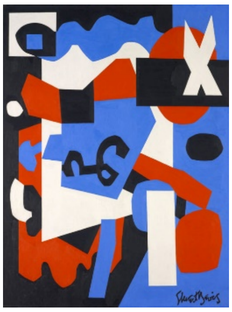

Stuart Davis, Ready to Wear, 1955

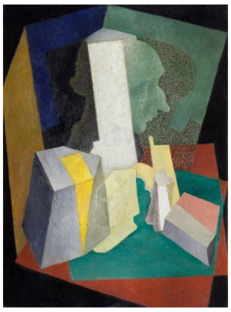

Diego Rivera, Composition with Bust, 1916

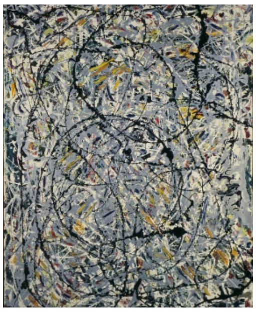

Jackson Pollock, Watery Paths, 1947

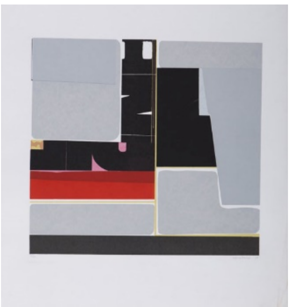

Fernando Garcia Ponce, Composición en gris, 1974

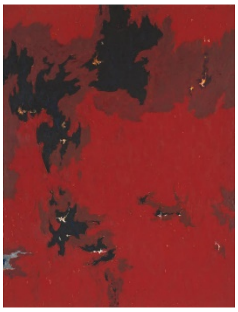

Clyfford Still, PH-385, 1949

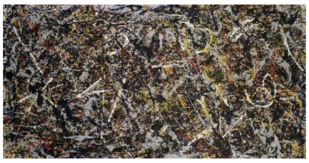

Jackson Pollock, Alchemy, 1947

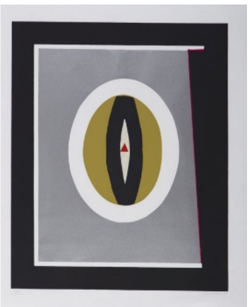

Fernando Garcia Ponce, Óvalo, 1974

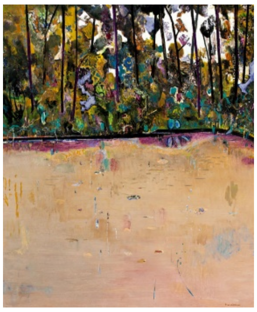

Fred Williams, Forest Pond, 1974

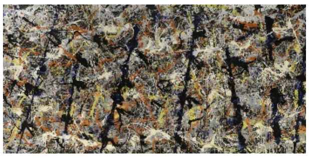

Jackson Pollock, Blue Poles, 1952

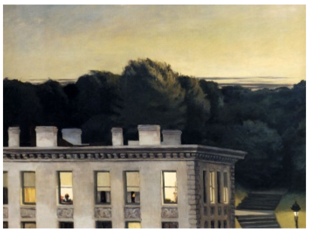

Edward Hopper, House at Dusk, 1935

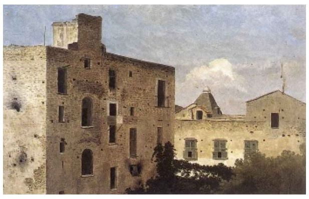

Thomas Jones, House in Naples, 1782

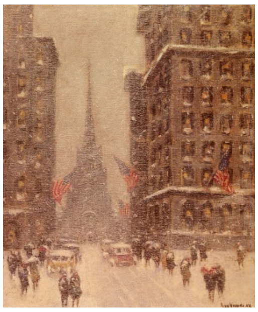

Guy C. Wiggins, Trinity Church, Wall Street, 1920

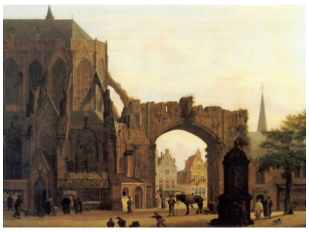

Jan Hendrick Verheyen, Figures Near a Church, 1810

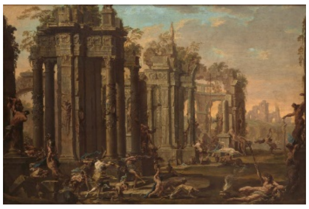

Alessandro Magnasco, Bacchanal, 1720-1730

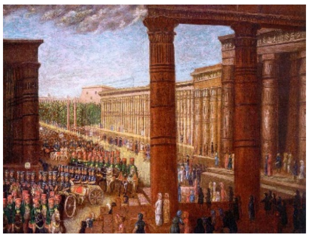

Erastus Salisbury Field, Pharaoh's Army Marching, 1865-1880

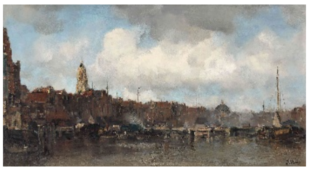

Jacobus Hendricus Maris, A View of a Harbor Town, 1880

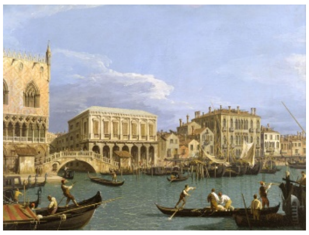

Giovanni Antonio Canal (Canaletto), View of the Riva degli Schiavoni, Venice, 1735-1739

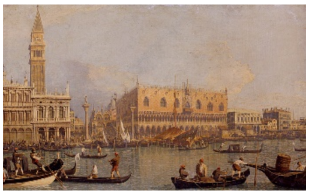

Giovanni Antonio Canal (Canaletto), Ducal Palace, Venice, 1755

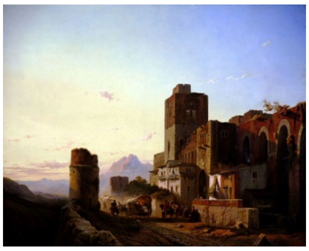

François-Antoine Bossuet, Spanish Town

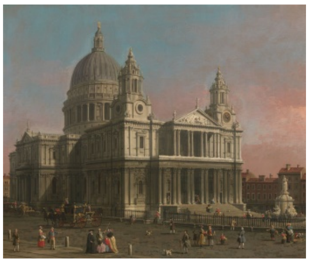

Giovanni Antonio Canal (Canaletto), St. Paul's Cathedral, 1754

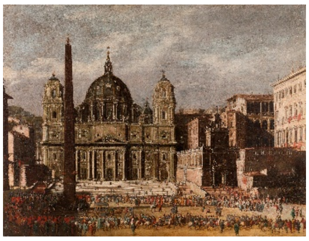

Viviano Codazzi, St Peter's, Rome Date,1630

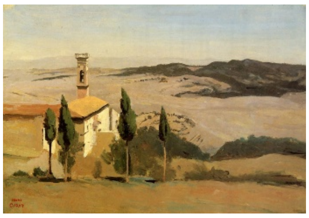

Jean Baptiste Camille Corot, Church and Bell Tower, 1834

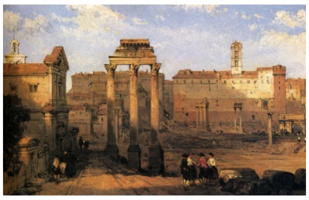

David Roberts, The Forum Rome, 1859

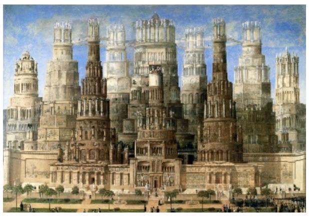

Erastus Salisbury Field, Historical Monument of the American Republic, 1867

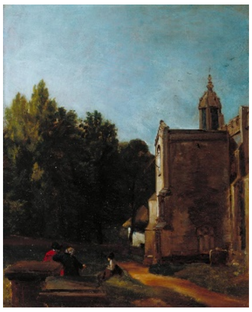

John Constable, The Church Porch, East Berholt, 1809

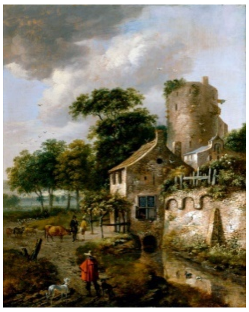

Roelof Van Vries, Landscape with a Tower, 1680

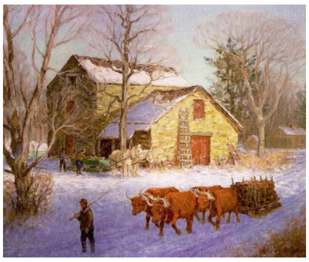

Winfield Scott Clime, American, 1881-1958

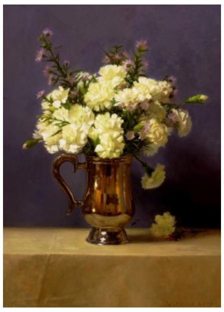

Patricia Watwood, Carnations and September, 2000

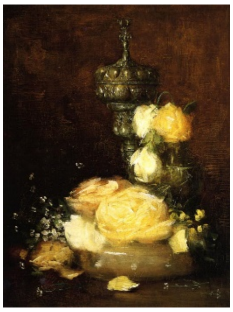

Julian Alden Weir, Silver Chalice with Roses, 1882

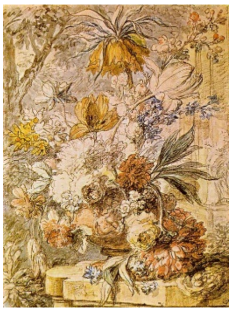

Jan Van Huysum, Vase with Flowers, 1726

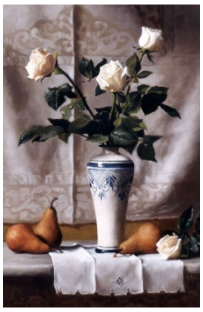

Maureen Hyde, Still Life with White Roses

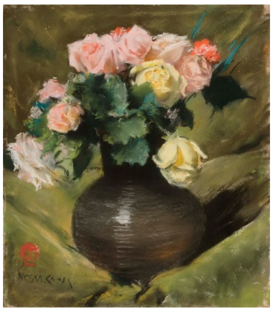

William Merritt Chase, Roses, 1883

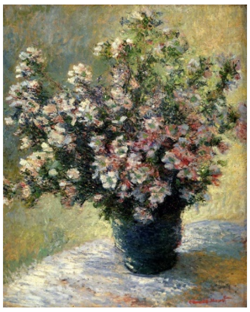

Claude Monet, Vase of Flowers, 1882

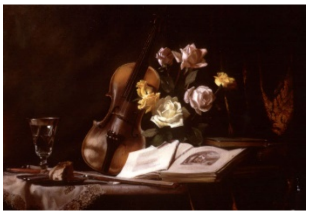

Maureen Hyde, Still Life with Violin and Roses,

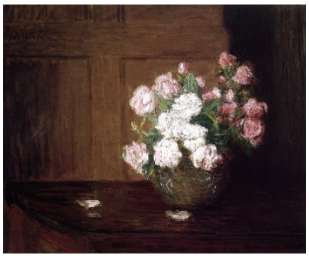

Julian Alden Weir, Roses in a Silver Bowl on a Mahogany Table, 1890

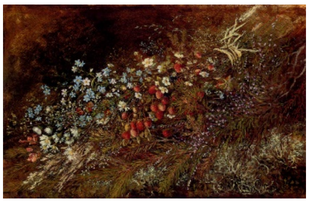

Olga Wisinger-Florian, Bouquet of Summer Fruits and Flowers, 1844-1926

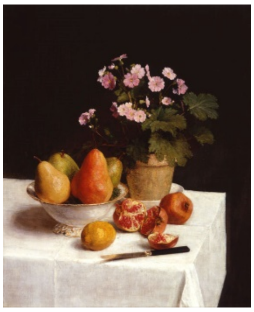

Henri Fantin-Latour, Still life (primroses, pears and promenates), 1869

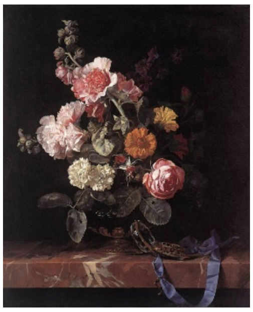

William van Aelst, Vase of Flowers with Watch, 1656

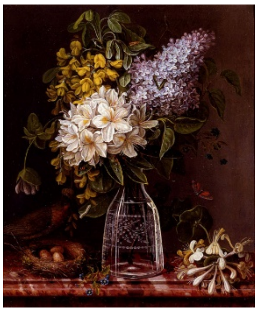

Theodore Jozef Sax, Still Life of Lilacs and Other Flowers, 1822

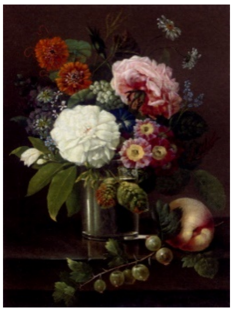

Johan Carl Smirsch, Roses, Marigolds, Daisies, Primroses and Other Summer Blooms in a Glass, 1801-1969

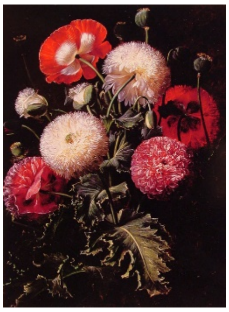

Joahn Laurentz Jensen, Still Life with Pink Red and White Poppies, 1800-1856

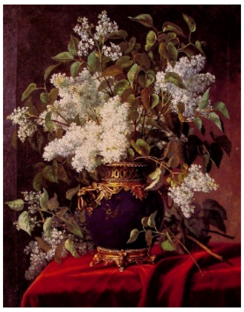

Jean Capeinick, White Lilacs, 1838-1890

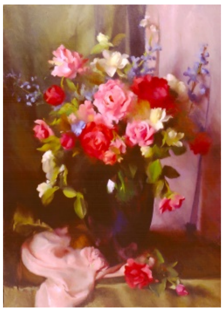

Mary Minifie, Roses and Peonies II, 1951-

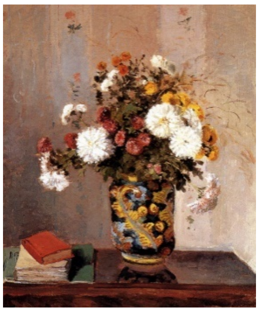

Camille Pissarro, Chrysanthemums in a Chinese Vase, 1873

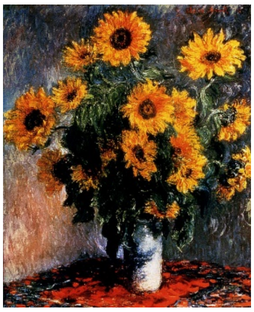

Claude Monet, Sunflowers, 1881

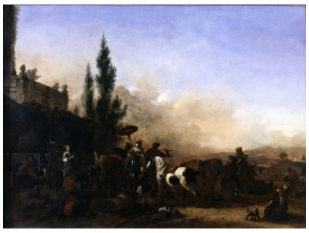

Philips Wouwerman, The Return from Hawking, 1660-1665

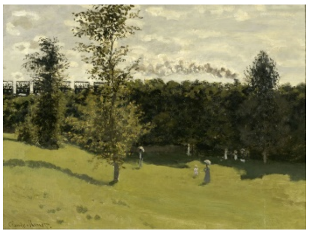

Claude Monet, Train in the Countryside, 1870

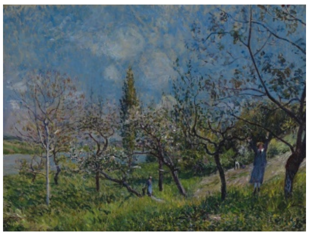

Alfred Sisley, Orchad in Spring, 1881

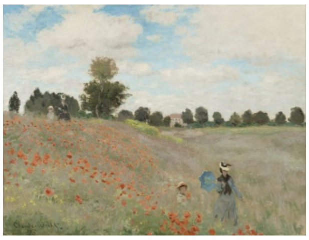

Claude Monet, The Poppy Field, 1873

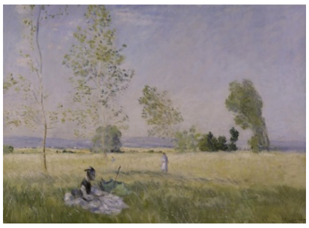

Claude Monet, Summer, 1874

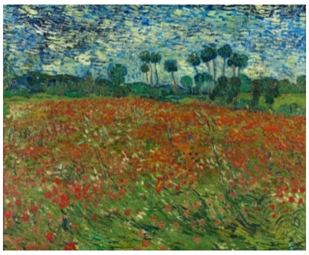

Vincent Van Gogh, Field of Poppies, 1890

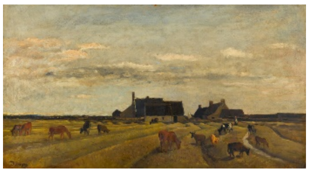

Charles-François Daubigny, Farm at Kerity, Brittany, 1817-1878

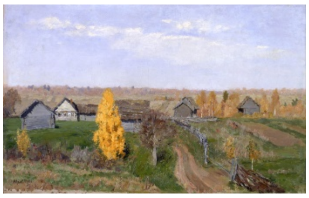

Isaac Levitan, Golden Autumn, Slobodka, 1889

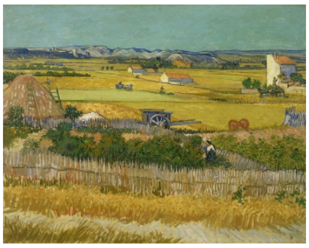

Vincent Van Gogh, The Harvest, 1888

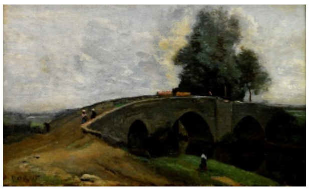

Jean Baptiste Camille Corot, Le vieux pont, 1796-1875

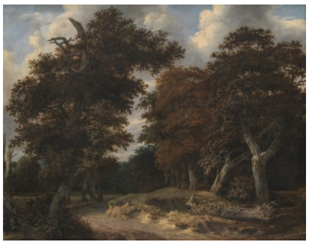

Jacob van Ruisdael, Road through an Oak Forest, 1646

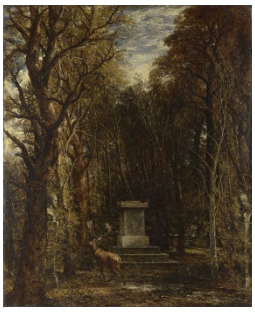

John Constable Cenotaph to the Memory of Sir Joshua Reynolds, 1833-1836

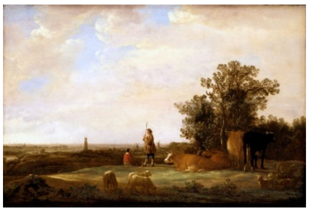

Aelbert Cuyp, View on a Plain, 1644

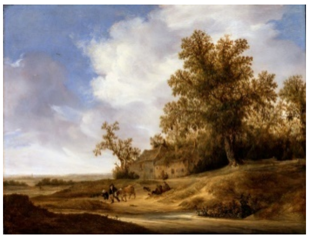

Jakob Van Moscher, A Road Near Cottages, 1655

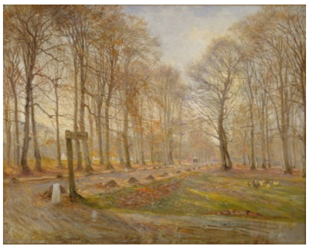

Theodor Phillipsen, Late Autumn Day in the Jægersborg Deer Park, North of Copenhagen, 1886

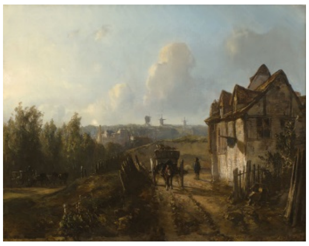

Johan Barthold Jongkind, View on Monmartre, 1850

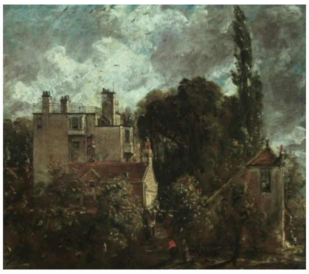

John Constable, The Grove, or Admiral's House, Hampstead, 1821

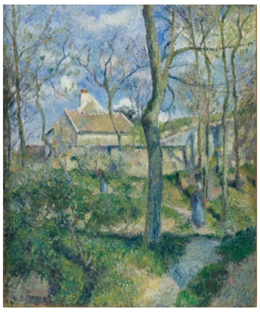

Camille Pissarro, the Path to Les Pouilleux, Pontoiuse, 1881

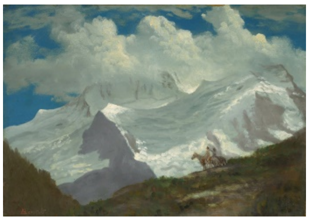

Albert Bierstadt, In the Rockies, 1863

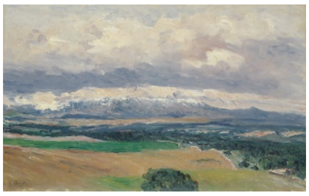

Aureliano de Beruete, View of The Guadarrama Mountains from El Plantío, 1901

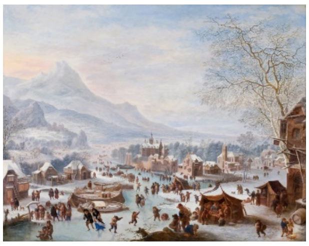

Jan Griffier, Winter Scene with Skaters, 1652-1718

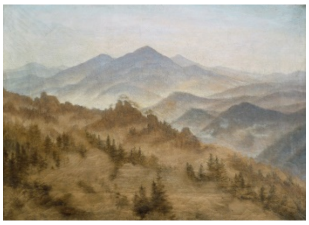

Caspar David Friedrich, Landscape with the Rosenberg in the Bohemian Mountains, 1835

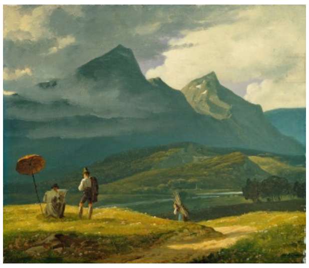

Wilhelm Bendz, Mountain Landscape, 1831

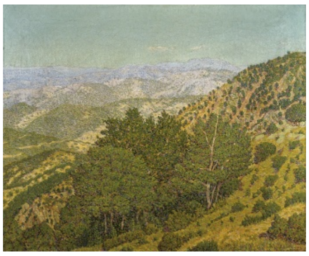

Marià Pidelaserra, Mountains from Montseny. A Calm Day in the Morning, 1903

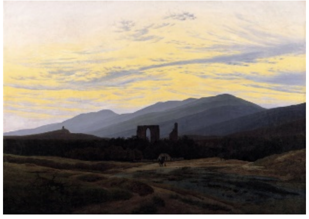

Caspar David Friedrich, The ruins of Eldena in the Giant Mountains, 1830-1834

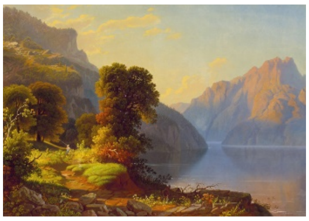

George Caleb Bingham, View of a Lake in the Mountains, 1856-1859

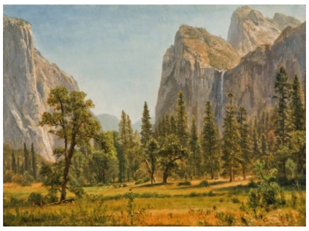

Albert Bierstadt, Valley of the Yosemite, 1864

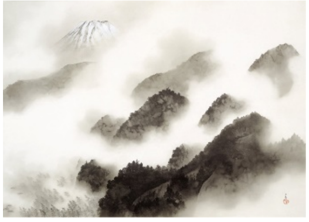

Yokoyama Taikan, Mountain after a Shower, 1940

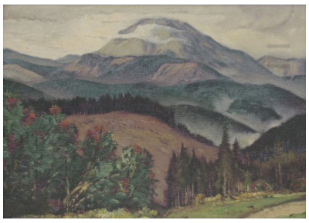

Martin Benka, Choč Mountain, 1925-1930

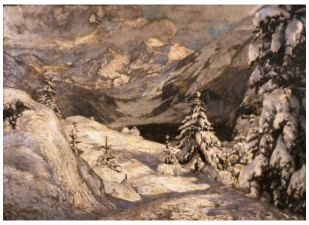

Filippo Carcano, In Midwinter, 1909

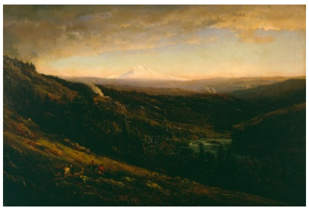

William Keith, Mountain Hood from Sandy River, 1870-1880

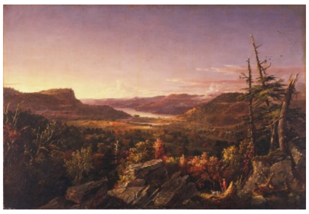

Jasper Francis Cropsey, View of Greenwood Lake, New Jersey, 1845

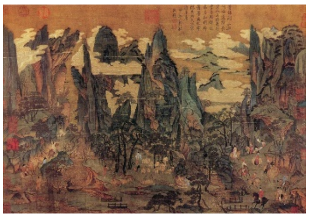

Li Zhaodao, Emperor Xuanzong's Flight to Shu, 907

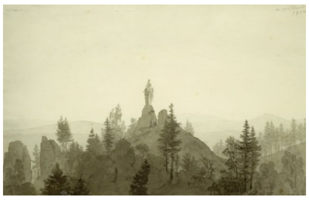

Caspar David Friedrich, Statue of the Madonna in the Mountains, 1804

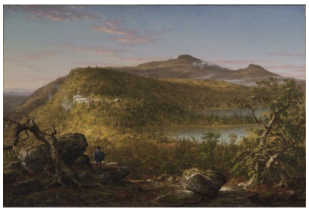

Thomas Cole, A View of the Two Lakes and Mountain House, Catskill Mountains, Morning, 1844

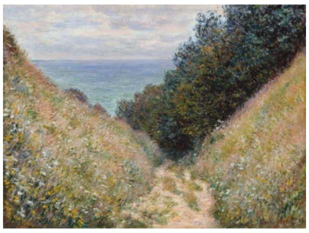

Claude Monet, Road at La Cavée, Pourville, 1882

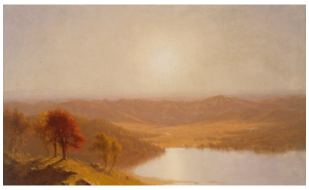

Sanford Robinson Gifford, A View from the Berkshire Hills, near Pittsfield, Massachusetts, 1863

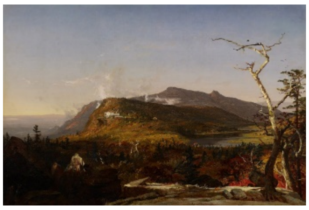

Jasper Francis Cropsey, Catskill Mountain House, 1855

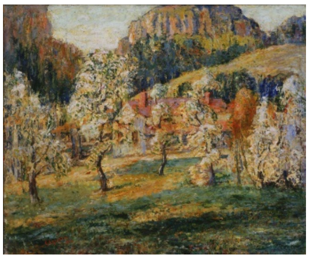

Ernest Lawson, May in the Mountains, 1919

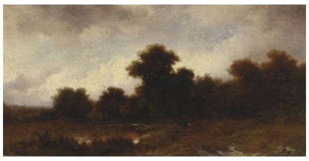

Remigius Adrianus Haanen, Wetland Landscape, 1874

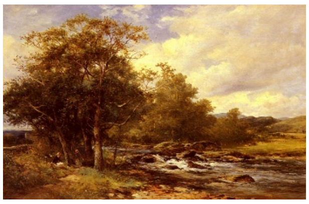

David Bates, Resting Beside a River, 1900

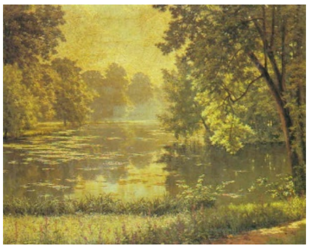

Henri Biva, A Wooded River Landscape, 1848-1929

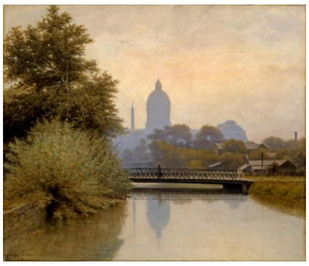

Richard Gruelle, The Canal Morning Effect, 1894

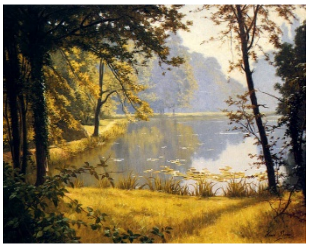

Henri Biva, A Lily Pond, 1848-1929

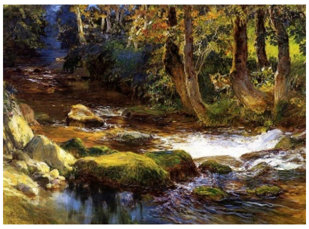

Frederick Arthur Bridgman, River Landscape with Deer, 1847-1928

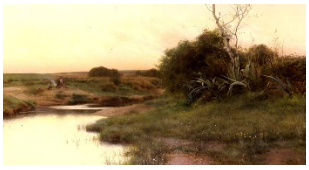

Emilio Sanchez-Perrier, On The River's Edge At Dusk, 1855-1907

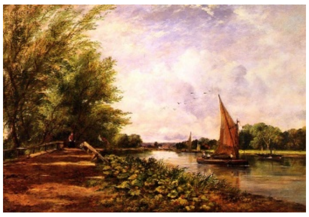

Frederick Waters Watts, The Riverbank, 1800-1862

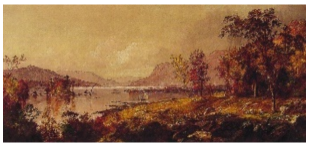

Jasper Francis Cropsey, Greenwood Lake in September, 1823-1900

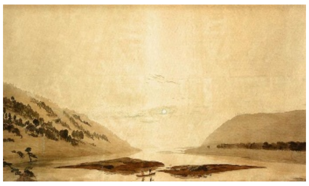

Caspar David Friedrich, Mountainous River Landscape Mroning, 1830

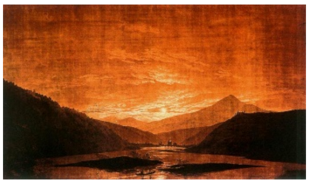

Caspar David Friedrich, Mountainous River Landscape Night Version, 1830

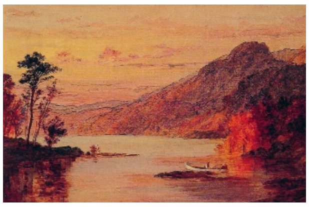

Jasper Francis Cropsey, Lake Scene Catskill Mountains, 1899

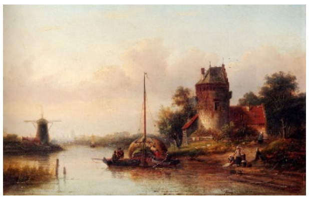

Jan Jacob Spohler, A River Landscape in Summer with a Moored Haybarge by a Fortified Farmhouse, 1811-1866

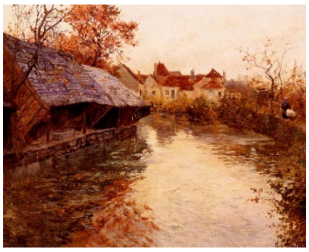

Fritz Thaulow, A Morning River Scene, 1891

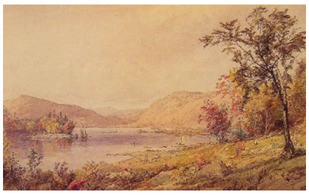

Jasper Francis Cropsey, Greenwood Lake, 1823-1900

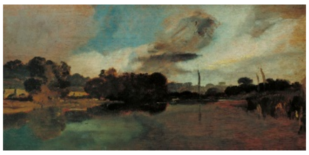

J oseph Mallord William Turner, Walton Reach, 1805

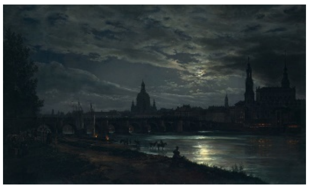

Johan Christian Claussen Dahl, View of Dresden at Full Moon, 1839

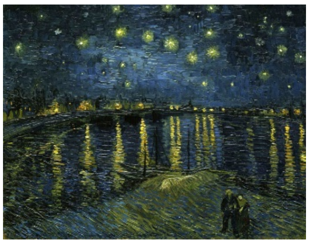

Vincent Van Gogh, Starry Night over the Rhône, 1888

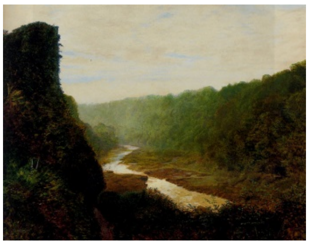

John Atkinson Grimshaw, Landscape with Winding River, 1868

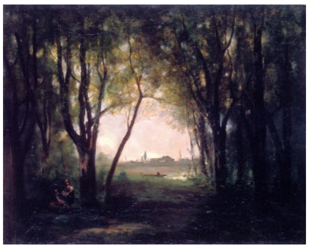

Jean Baptiste Camille Corot, Landscape with Lake, 1860-1873

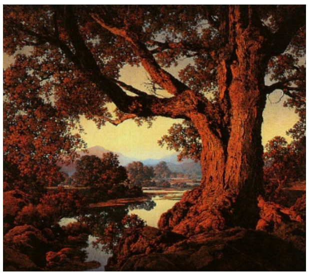

Maxfield Parrish, Riverbank in Autumn, 1938

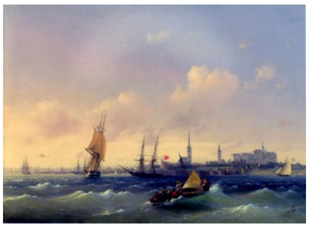

Ivan Aivazovsky, Reval, 1845

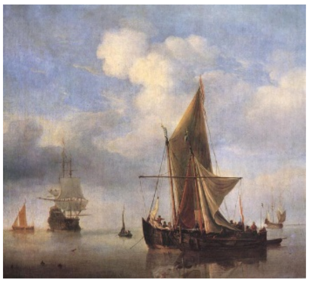

Willem van de Velde the Younger, Calm Sea, 1633-1707

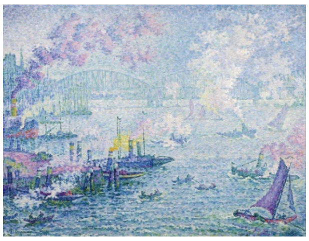

Paul Signac, The Port of Rotterdam, 1907

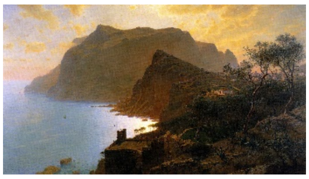

William Stanley Haseltine, The Sea from Capri, 1875

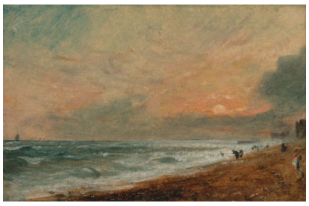

John Constable, Hove Beach, 1824-1828

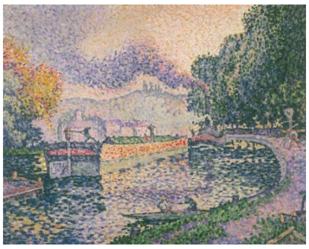

Paul Signac, The Tugboat, Canal in Samois, 1901

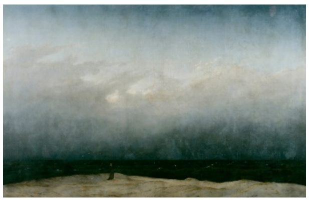

Casper David Freidrich, The Monk by the Sea, 1808-1810

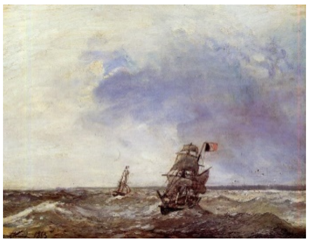

Johan Barthold Jongkind, Ships at Sea, 1864

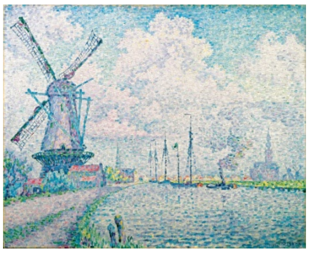

Paul Signac, Canal of Overschie, 1901

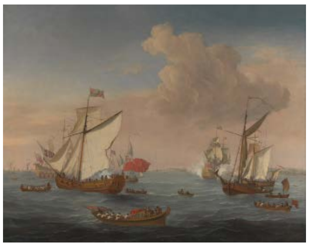

Isaac Sailmaker, Ships in the Thames Estuary near Sheerness, 1707

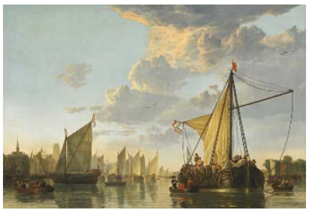

Aelbert Cuyp, The Maas at Dordrecht, 1650

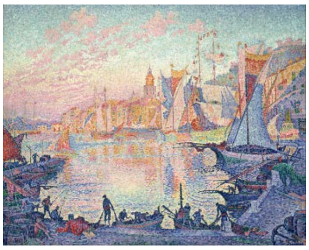

Paul Signac, The Port of Saint Tropez, 1901

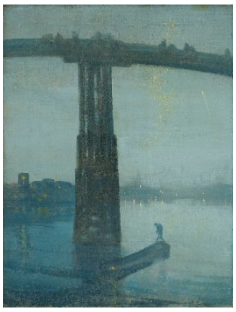

James Abbott McNeill Whistler, Nocturne: Blue and Gold – Old Battersea Bridge, 1872-1875

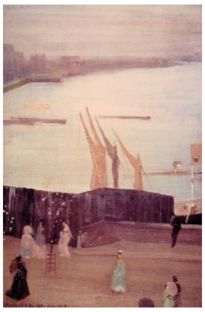

James McNeill Whistler, Variations in Pink and Grey: Chelsea, 1871

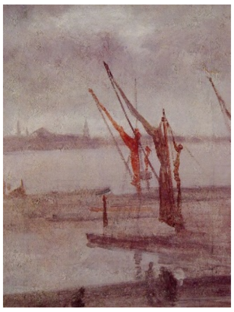

James Abbott McNeill Whistler, Grey and Silver: Chelsea Wharf, 1875
